# Pricing of cyber insurance premiums using a Markov-based dynamic model with clustering structure

**DOI:** 10.1371/journal.pone.0258867

**Published:** 2021-10-26

**Authors:** Yeftanus Antonio, Sapto Wahyu Indratno, Suhadi Wido Saputro

**Affiliations:** 1 Statistics Research Division, Institut Teknologi Bandung, Bandung, West Java, Indonesia; 2 University Center of Excellence on Artificial Intelligence for Vision, Natural Language Processing & Big Data Analytics (U-CoE AI-VLB), Institut Teknologi Bandung, Bandung, West Java, Indonesia; 3 Combinatorial Mathematics Research Division, Institut Teknologi Bandung, Bandung, West Java, Indonesia; Universita degli Studi di Catania, ITALY

## Abstract

Cyber insurance is a risk management option to cover financial losses caused by cyberattacks. Researchers have focused their attention on cyber insurance during the last decade. One of the primary issues related to cyber insurance is estimating the premium. The effect of network topology has been heavily explored in the previous three years in cyber risk modeling. However, none of the approaches has assessed the influence of clustering structures. Numerous earlier investigations have indicated that internal links within a cluster reduce transmission speed or efficacy. As a result, the clustering coefficient metric becomes crucial in understanding the effectiveness of viral transmission. We provide a modified Markov-based dynamic model in this paper that incorporates the influence of the clustering structure on calculating cyber insurance premiums. The objective is to create less expensive and less homogenous premiums by combining criteria other than degrees. This research proposes a novel method for calculating premiums that gives a competitive market price. We integrated the epidemic inhibition function into the Markov-based model by considering three functions: quadratic, linear, and exponential. Theoretical and numerical evaluations of regular networks suggested that premiums were more realistic than premiums without clustering. Validation on a real network showed a significant improvement in premiums compared to premiums without the clustering structure component despite some variations. Furthermore, the three functions demonstrated very high correlations between the premium, the total inhibition function of neighbors, and the speed of the inhibition function. Thus, the proposed method can provide application flexibility by adapting to specific company requirements and network configurations.

## Introduction

Currently, cyber risk management using cyber insurance is increasingly needed. Cyber risk is a type of operational risk that arises from the execution of cyberspace activities, posing a threat to information assets, information and communication technology (ICT) resources, and technological assets [1]. This risk has rapidly changed the cyber insurance landscape due to technological advances and continues to increases every year [2]. During the coronavirus pandemic, there has been an increase in cyber-attacks targeting vulnerable sectors and, thus, the cyber-attack success rate [3]. Global cybercrime costs are expected to rise 15% each year over the next five years reaching US $10.5 trillion annually by 2025, up from US $3 trillion in 2015, representing the most significant transfer of economic capital in history [4]. Cyber insurance markets and industries are also continuing to expand. The global cyber insurance market was valued at US $4.85 billion in 2018, according to Allied Market Research, and is expected to reach US $28.60 billion by 2026 [5]. According to RBC Capital Markets, the global cyber insurance market was worth $6 billion in 2019 and will be worth $15 billion by 2022 [6].

Cyber risk and cyber insurance have been a concern of many researchers in recent years. Cyber threats can be classified based on their frequency, severity, and dependence structure [7]. Based on the network structure, cyber insurance modeling can be divided into nonnetwork models and network models. Several mathematical models were introduced into the nonnetwork model. Farkas et al. [8] proposed the generalized Pareto regression tree to identify criteria for evaluating and classifying cyber claims. Other mathematical models are the beta-binomial model by Böhme and Schwartz [9], copula by Herath and Herath [10], collective risk theory by Mukhopadhyay et al. [11], and extreme value theory by Eling and Schnell [12]. These models, in general, use data on operational risk from ICT assets, cyber incidents, loss, system updating, monitoring, and security.

Another approach involves a network model in cyber risk estimation. Fahrenwaldt et al. [13] suggest the pricing of cyber insurance contracts in a network model. The authors developed the first insured loss mathematical model generated by infectious cyber threats. They used a susceptible-infectious-susceptible (SIS) network process [14–16] for a cyber infection model. An undirected network represents risk dependencies where each node could be a company, computer system, or a single device, and each edge or link is a transmission line in the network. The insured network structure substantially affects the loss numerical study on homogeneous, clustered and star-shaped networks. The results showed that the network topology was an essential element for pricing cyber insurance contracts and cyber risk management.

Under the assumption of a tree-based local area network (LAN) topology, Jevti and Lanchier [17] present a structural model of aggregate cyber loss distribution for small- and medium-sized businesses. Hua and Xu [18] proposed a risk-spreading and recovering algorithm for generating synthetic data. To account for the uncertainty of random large-scale network topology, they adopted a scale-free network framework. Xu and Hua [19] considered the network model through Markov, non-Markov, and copula processes. In the area of cyberattacks, Markov-based models are frequently utilized. Along with the epidemic model, this model can detect abnormalities caused by cyber threats under noise restriction [20]. Some researchers use wavelet analysis [21] for cybersecurity models, such as the detection of attack anomalies in network traffic [22, 23] or disease spread models [24]. In the Markov-based cyber insurance model, the generalized SIS process (*ε*-SIS) [25] describes the virus spread dynamics in a network. Xu and Hua [19] used cost functions for two types of losses: data damage losses and system downtime losses. Insurance premiums are calculated from a microlevel perspective using the standard deviation premium principle and the utility principle. A small ten-node network was used as a case study, and an Enron e-mail network was used as an application of the models.

The results of cyber insurance research with network models show the importance of network structure in cyber risk estimation. Additionally, the importance of generating synthetic data from infection and recovery dynamics based on certain assumptions is shown as the solution to current cyber incident data limitations. Thus, network characteristics and metrics are critical considerations in modeling the dynamics of virus spread. However, experimental results by Xu and Hua [19] only showed the strong influence of the degree of a node in a network on cyber losses and premiums. To confirm this, we conducted a study on the regular graph using the Markov model and obtained similar results [26]. The degree of a node can only explain the number of neighbors but has not described the relationship between neighbors. Two or more nodes with the same degree can have different neighboring connection structures. The structure between neighbors of a node can be described by a network metric called the clustering coefficient, which is a clustering coefficient for how closely nodes in a graph cluster together [27]. In other words, the clustering coefficient can explain the clustering structure of a network.

Several experiments have shown the influence of the clustering coefficient on disease transmission [28–31]. Assuming that social networks have a high community structure and clustering coefficient, Wu and Liu [32] proposed a new model to study their influence on epidemics. According to their findings, the degree of the community determines the spread of epidemics in community networks. In contrast, an increase in the clustering coefficient reduces the epidemic spread efficiency for a community with a fixed degree. Using the SIS process, Bo Song et al. [33] concluded the same thing that in a homogeneous network (same degree for each node), clustering could inhibit epidemics. Conversely, there is no inhibiting effect during infection in heterogeneous networks. However, no one has created a model at the individual level that can explain the dynamic process of infection to the status of an individual [34].

This study proposes a Markov-based model with the network structure effect, namely, the *ε*-SIS model with a clustering coefficient factor for cyber insurance pricing. We incorporate the coefficient clustering function [32, 33] into the transition probability of the Markov model or *ε*-SIS process [25]. Cyber insurance rates are calculated using the cost function based on two types of losses by Xu and Hua [19]. In contrast, the simulation process is run using a modified Markov-based simulation with different infection rates. In previous work, we used the average degree factor as a matrix of the network in a compartment SIS process [35]. We propose a modified Markov-based algorithm with different rates at the individual-level *ε*-SIS model to generate synthetic cyber-attack data in this study. This algorithm is a modification of the individual-level SIS process algorithm with homogeneous rates. The procedure was implemented through a case study on a regular (homogeneous) network using random regular graph sampling [36, 37]. Furthermore, the regular graph’s theoretical background and its relationship to the local clustering effect are also presented in this paper. Moreover, the findings are validated by implementation on a real network (large network).

The remainder of this paper is developed as follows. Materials and methods discusses the concepts and methods used for rate-making using a Markov-based model with a clustering structure. The main results and findings presented in Results and discussion include regular graph theory and clustering coefficients. Results and discussion also offers a discussion of the findings of a regular and email communication network. Conclusions and future work are presented in Conclusion.

## Materials and methods

This section discusses the theories and simulation methods used for cyber insurance pricing with a clustering structure factor. These are related to the definition of clustering coefficients and how this metric defines the Markov-based model’s infection rate, random regular graphs, and simulations using the modified Markov-based simulation.

### Clustering coefficient

Our model is an individual-level model where a node’s tendency to have a clustering structure depends on a metric known as the local clustering coefficient. Let an undirected graph *G* = (*V*, *E*) be a representation of a network where *V* is a set of vertices (nodes) and *E* is a set of edges (links). A link (*u*, *v*) ∈ *E* connects node *u* ∈ *V* and node *v* ∈ *V*. The set of neighbors of node *v* is denoted by *N*(*v*) = {*u*;(*v*, *u*) ∈ *E* ∧ (*u*, *v*) ∈ *E*}. Hence, the cardinality of *N*(*v*), also known as the degree of node *v*, expresses the number of neighbors of node *v* and can be written as |*N*(*v*)| = *k*_*v*_, where *k*_*v*_ is the degree of node *v*. A clique of three nodes {*u*, *v*, *w*}, where (*u*, *v*), (*u*, *w*), (*v*, *w*) ∈ *E* are links that connect all three nodes, is a triangle in a network *G* [38]. Let *T*(*v*) = |{(*u*, *w*);*w*, *u* ∈ *N*(*v*), (*u*, *w*) ∈ *E*}| be the number of triangles formed with the center at node *v*. The local clustering coefficient for node *v* is defined as

$$\begin{array}{c} C_{v}=\{\begin{array}{cc} \frac{2T(v)}{k_{v}\left(k_{v}-1\right)}=\frac{2|\{(u,w);w,u\in N(v),(u,w)\in E\}|}{k_{v}\left(k_{v}-1\right)} & \text{if}\;k_{v}>1\\ 0 & \text{if}\;k_{v}\leq 1 \end{array}. \end{array}$$ (1)

**Fig 1:**
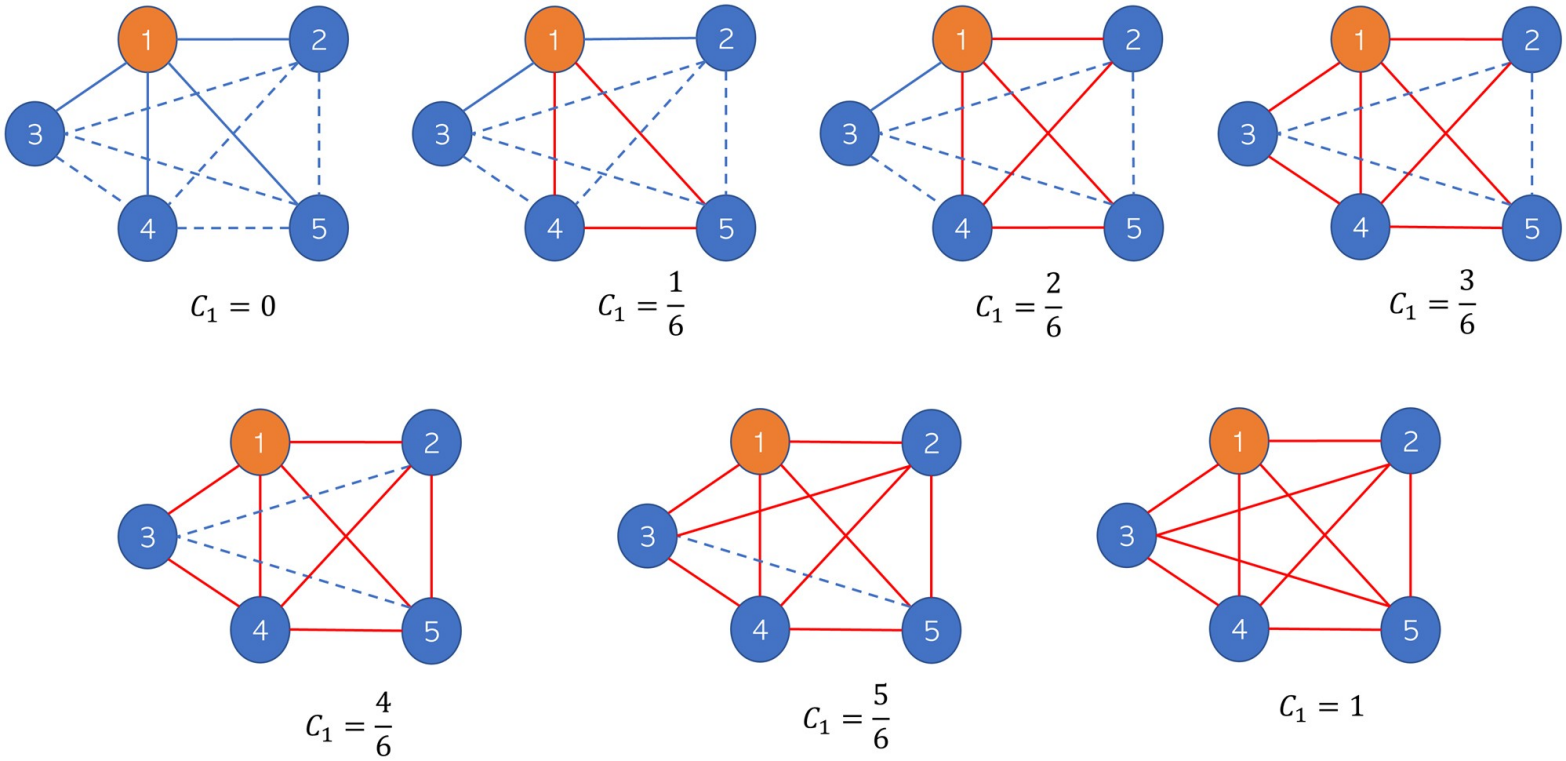
Local clustering coefficient of node 1. Possible local clustering coefficient at node 1 (orange node or *C*_1_) with degrees *k*_1_ = 4 on an undirected graph. The blue dashed lines represent the possible connections between the neighbors, and the red solid lines represent the triangles between the neighbors of node 1.

In terms of the relative density of connections in its neighborhood, it determines how connected its neighborhood is to a complete network. Thus, this metric measures the proportion of the number of triangles with the center at node *v* compared to the number of triangles between the neighbors of node *v* if all the neighbors are connected (complete network), namely, (kv2)=kv(kv-1)2. For example, Fig 1 illustrates the difference in the local clustering coefficient values at node 1 (*C*_1_). Node 1 has the same degree *k*_*v*_ = 4 for each structure. However, the relationship between its neighbors is different, which causes the local clustering coefficient value of node 1 to be different. In this case, the set of possible clustering coefficients for node 1 is $\left\{0,\frac{1}{6},\frac{2}{6},\frac{3}{6},\frac{4}{6},\frac{5}{6},1\right\}$. We have $\frac{k_{v}\left(k_{v}-1\right)}{2}$ possible pairs between neighbors and zero if no neighbors are connected. Fig 1 shows the network structure of each possible clustering coefficient. Thus, we can conclude that a node with the same degree can have different clustering coefficient values. By adding the clustering coefficient factor to the epidemic model, we can characterize the dynamics of the virus spread based on the structure between neighbors.

### Regular graph

A regular graph with degree *k* denoted by *k*-regular graph is a graph *G* = (*V*, *E*) where the degree of each node is the same, namely, *k*_*v*_ = *k* for every *v* ∈ *V*. In other words, each node in graph *G* has the same number of neighbors. Several graph theories are needed to determine the existence of a *k*-regular graph.

**Lemma 1** (The handshaking lemma [39]). *In any graph* *G* = (*V*, *E*) *where* |*E*| = *m*, *the sum of all degrees of node v* ∈ *V* *or* *deg*(*v*) *is twice the number of links and can be written as*
  $$\begin{array}{c} \sum_{v\in V}deg\left(v\right)=2m \end{array}$$ (2)
**Lemma 2** ([39]). *Graph G* = (*V*, *E*) *has an even number of nodes with odd degrees*.
Lemma 1 and Lemma 2 are met for all *G* = (*V*, *E*). Since the *k*-regular graph is a subset of *G* = (*V*, *E*), the following result is obtained:
**Corollary 1**. *A regular graph has an even number of nodes with odd degrees*.
**Lemma 3** (The existence of a regular graph). *The sufficient and necessary conditions for the existence of a k-regular graph with the order n are n* > *k* + 1 *and nk even*.

*Proof*. The maximum edge (link) of a graph with the order *n* is in a complete graph (n2)=n(n-1)2 and the order is *n* − 1. Thus, *k* = *n* − 1 or *n* = *k* + 1. This condition is the *n* minimum for a special *k*. Additionally, note that if a regular graph is of the order *n*, then the number of sides is $\frac{nk}{2}$; thus, *nk* must be even.

$$\begin{array}{c} \sum_{v\in V}deg\left(v\right)=2m\Leftrightarrow nk=2m\Leftrightarrow m=\frac{nk}{2} \end{array}$$ (3)

Therefore, for odd *n*, the regular graph is defined only for even *k*. Theoretical foundations for regular graphs are essential for the results and discussion sections to adequately describe the influence of clustering coefficients on regular graphs.

### Risk model and rate making theory

This study considers the cyber risk model by Xu and Hua (2019) [19]. This risk model uses two types of threats faced by each node: (1) threats from outside the network (for example, infection because node *v* was attacked or the user visited a malicious site) and (2) threats from within the network (e.g., infected node *v* attacking its neighbors). Assume that if a node is infected, it can be repaired and returned to a safe status but is still vulnerable to reinfection.

Suppose a cyberattack occurs on a network represented by an undirected graph *G* = (*V*, *E*) where *V* is a set of nodes, and *E* is a set of edges (links). Transmission on this network occurs via link (*u*, *v*) ∈ *E* so that node *u* and node *v* can attack each other. The number of nodes on the network is denoted by *N* = |*V*|. The degree of a node is the number of links associated with a node. The degree of node *v* is denoted by *deg*(*v*). An undirected graph *G* = (*V*, *E*) can be written into the adjacency matrix **A** = (*a*_*uv*_) where

$$\begin{array}{c} a_{uv}=\{\begin{array}{cc} 1, & \;\text{if}\;a_{uv}\in E\\ 0, & \;\text{if}\;a_{uv}\notin E \end{array} \end{array}$$ (4)

Let there be *N* computers or devices such that *v* ∈ 1, 2, ⋯, *N*. The status of the network at time *t* can be written as the vector **I**^⊤^(*t*) = (*I*_1_(*t*), *I*_2_(*t*), ⋯, *I*_*N*_(*t*)), where *I*_*v*_(*t*) = 1 when node *v* is infected at times *t* and *I*_*v*_(*t*) = 0 if node *v* is secure (but vulnerable to attack) at times *t* to *v* = 1, 2, ⋯, *N*. The infection probability vector is denoted by **p**^⊤^(*t*) = (*p*_1_(*t*), *p*_2_(*t*), ⋯, *p*_*N*_(*t*)), where *p*_*v*_(*t*) = *P*(*I*_*v*_(*t*) = 1) for *v* = 0, 1, 2, ⋯, *N*.

**Fig 2:**
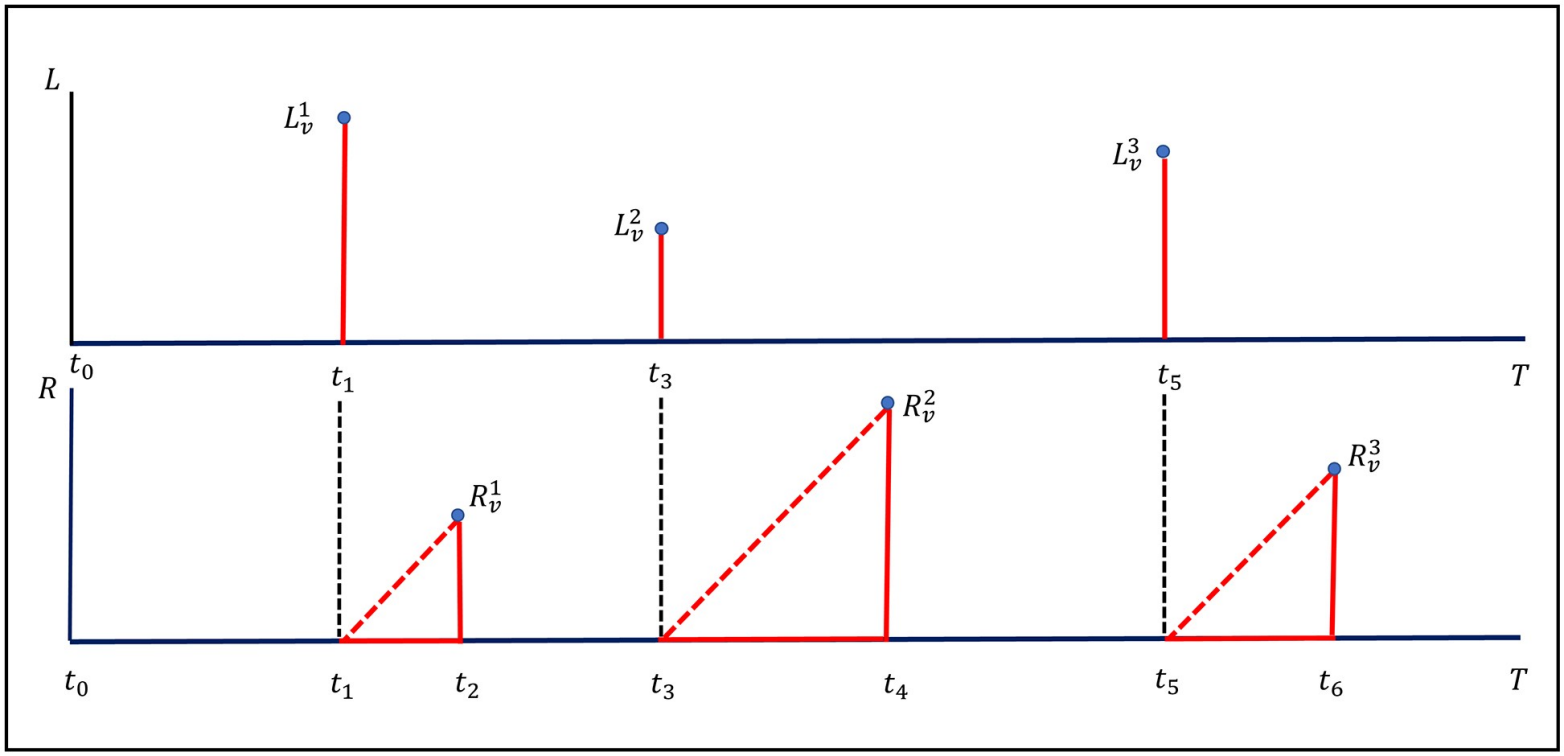
Losses faced by a computer (node). Two types of losses are faced by node *v* in a network during the time interval [*t*_0_, *T*]. *L* is losses caused by data damage, and *R* is losses caused by system downtime.

Fig 2 describes two types of risk that occur at a node in a network. Suppose that at the time of observation [0, *T*], a node *v* is safe at time *t*_0_ and then has three infections, namely, at times *t*_1_, *t*_3_, and *t*_5_. Such an infection can cause two types of losses:

1. Losses caused by infection, such as data corruption, extortion, information theft, hacking, denial of service and third-party fees.
2. Losses caused by the length of time to repair the computer (system downtime).

At the first time *t*_1_ infection caused data corruption or damage at node *v* is $L_{v}^{1}$ and loss due to system downtime is $R_{v}^{1}$. The losses for the second infection are $L_{v}^{2}$ and $R_{v}^{2}$, respectively, and the losses for the third infection are $L_{v}^{3}$ and $R_{v}^{3}$, respectively. Thus, the total loss up to time *t* can be written as

$$\begin{array}{c} S_{v}\left(t\right)=\sum_{i=1}^{M_{v}\left(t\right)}\left[\mu_{v}\left(L_{v}^{i}\right)+\gamma_{v}\left(R_{v}^{i}\right)\right] \end{array}$$ (5)

where *M*_*v*_(*t*) is the number of infections from node *v* to time *t*, *μ*_*v*_(⋅) is the cost function due to infection and *δ*_*v*_(⋅) is the cost function corresponding to the length of time-to-repair. The total loss faced by the firm until *t* is

$$\begin{array}{c} S\left(t\right)=\sum_{v=1}^{N}S_{v}\left(t\right)=\sum_{v=1}^{N}\sum_{i=1}^{M_{v}\left(t\right)}\left[\mu_{v}\left(L_{v}^{i}\right)+\gamma_{v}\left(R_{v}^{i}\right)\right] \end{array}$$ (6)

Thus, the key quantity is how to obtain *M*_*v*_(*t*), which depends on the vector of network status up to time *t*, that is, **I**^⊤^(*t*). Network status vectors are obtained using a modified Markov-based model (in-homogeneous SIS) process with an inhibition function of the clustering coefficient.

### Modified Markov-based model

Wu and Liu (2008) [32] proposed a new model to study the effect of clustering coefficients on epidemics. According to their findings, the community level determined the spread of the virus in community networks. Conversely, an increase in clustering coefficients reduced the efficiency of epidemic spreading to a fixed community level. Using the SIS process, Bo Song et al. (2017) [33] concluded the same thing that in a homogeneous network (same degree for each node), clustering could inhibit epidemics. In contrast, there was no inhibitory effect during infection in the heterogeneous network. However, no one has yet created a model at the individual level that can explain a more specific dynamic process [34].

The clustering coefficient influences the infection rate for each node. Let the *f*(*C*_*v*_) function describe the effect of the high cluster on the epidemic spread speed at node *v*. With the same assumptions, the necessary conditions for *f*(*C*_*v*_) are

1. 0 < *f*(*C*_*v*_) < 1, and
2. *f*(*C*_*v*_) is a descending function that is $\frac{df(C_{v})}{dC_{v}}<0$.

**Fig 3:**
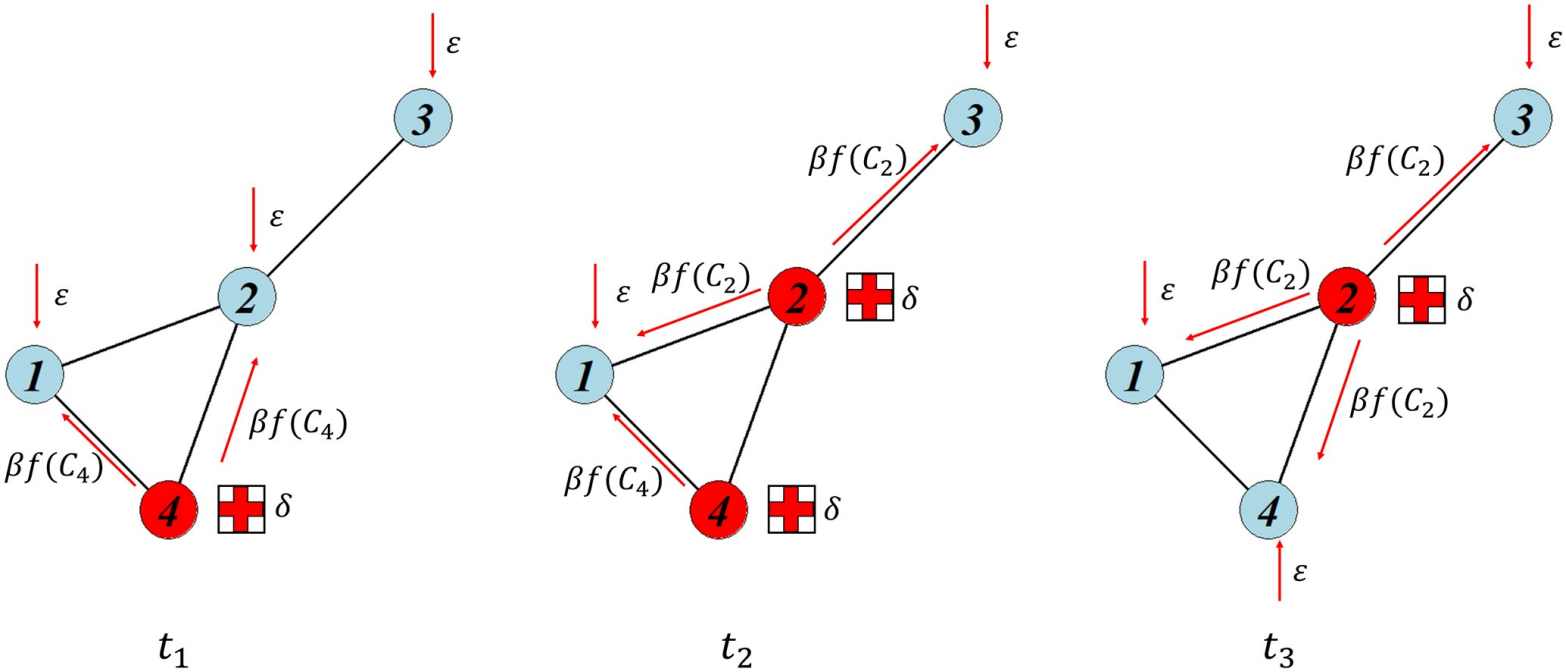
Dynamics of the modified Markov-based model. The dynamics of the infection and recovery processes of a network follow a modified *ε*-SIS model with local coefficient clustering factors *f*(*C*_*v*_) for time steps *t*_1_, *t*_2_, and *t*_3_. Red nodes indicate that the nodes are infected, and blue nodes indicate that the nodes are vulnerable at a certain time.

Fig 3 describes the process of this clustering function affecting the infection rate of each node. Thus, the transition probability can be written as:

$$\begin{array}{c} p_{v,xy}\left(h\right)=\{\begin{array}{cc} \left(\beta \sum_{j=1}^{N}f\left(C_{j}\right)a_{vj}I_{j}\left(t\right)+\varepsilon \right)h+o\left(h\right), & \text{if}\;x=0,y=1\\ \delta h+o(h), & \text{if}\;x=1,y=0 \end{array}. \end{array}$$ (7)

By supposing *β*_*j*_ = *βf*(*C*_*j*_), this process is a process of an in-homogeneous SIS model [40].

An in-homogeneous SIS model accommodates different infection rates for each node. Van Mieghem and Omic (2013) introduced an in-homogeneous SIS model [40]. The model adjusts the characteristics of different nodes in carrying out attacks, for example, the speed of the data transfer signal. If node *j* is infected at a particular time, it will attack its neighbors at the rate of *β*_*j*_.

Suppose that in an in-homogeneous SIS model, *β*_*j*_ is the infection rate for node *j*. If node *j* is infected, the time-to-infection of node *v* due to attack from node *j* is an exponential random variable with a mean equal to $\beta_{j}^{-1}$. The time it takes for node *v* to repair is an exponential random variable with a mean equal to $\delta_{v}^{-1}$. Likewise, the time-to-infection of node *v* due to external net factors is an exponential random variable with a mean of $\varepsilon_{v}^{-1}$. The following equation gives the transition probability.

$$\begin{array}{c} p_{v,xy}\left(h\right)=\{\begin{array}{cc} \left(\sum_{j=1}^{N}\beta_{j}a_{vj}I_{j}\left(t\right)+\varepsilon \right)h+o\left(h\right), & \text{if}\;x=0,y=1\\ \delta h+o(h), & \text{if}\;x=1,y=0 \end{array} \end{array}$$ (8)

where *I*_*j*_(*t*) is the status of node *j* at time *t* and the *β*_*j*_ attack rate of the infected neighbor of node *v*, i.e., node *j*. This model will be used to obtain the upper bound of infection probabilities and Monte Carlo simulations.

The dynamic equation for the infection probability from the in-homogeneous SIS model can be obtained with *N*-intertwined mean-field approximation (NIMFA) [41] as follows:

$$\begin{array}{c} \frac{dp_{v}\left(t\right)}{dt}=\sum_{j=1}^{N}\beta_{j}a_{vj}p_{j}\left(t\right)-\sum_{j=1}^{N}\beta_{j}a_{vj}p_{j}\left(t\right)p_{v}\left(t\right)-\left(\delta_{v}+\varepsilon_{v}\right)p_{v}\left(t\right)+\varepsilon_{v}. \end{array}$$ (9)

Another approximation uses the upper bound for the infection probabilities. Cator and Mieghem proved that

$$\begin{array}{c} E\left[I_{v}\left(t\right)I_{j}\left(t\right)\right]\geq E\left[I_{v}\left(t\right)\right]E\left[I_{j}\left(t\right)\right]. \end{array}$$ (10)

In other words, *I*_*v*_(*t*) and *I*_*j*_(*t*) are nonnegatively correlated for all finite graphs. These results lead to the upper bound for the infection probabilities, previously introduced for the *ε*-SIS model [19].

Upper bounds for infection probabilities are conservative estimates of the premium [19]. These upper bounds are obtained by solving the dynamic equation for the infection probabilities.

**Theorem 1**. *For the in-homogeneous SIS model with infection rate* *β*_*j*_ *for j* = 1, 2, ⋯, *N*, *recovery rate* *δ*_*v*_ = *δ* *and self-infection rate ε*_*v*_ = *ε*, *the upper bound of the infection probabilities are given by*

$$\begin{array}{c} p^{*}\left(t\right)=e^{\bar{Q}t}p^{*}\left(0\right)+{\bar{Q}}^{-1}\left[e^{\bar{Q}t}-I\right]\varepsilon \end{array}$$ (11)

*where* $\bar{Q}=diag(\frac{\delta }{\delta +\varepsilon })\;Adiag\left(\beta_{j}\right)-diag\left(\varepsilon +\delta \right)$, **ε**^*T*^ = (*ε*_1_, *ε*_2_, ⋯, *ε*_*N*_), and $e^{Qt}=\sum_{k=1}^{\infty }\frac{Q^{k}t^{k}}{k!}$.

*Proof*. The upper bound of dynamic infection probabilities in matrix and vector notations is given by

$$\begin{array}{c} \frac{dp(t)}{dt}\leq Adiag\left(\beta_{j}\right)p\left(t\right)-diag\left(p_{v}\left(t\right)\right)Adiag\left(\beta_{j}\right)p\left(t\right)-\left(\delta_{v}+\varepsilon_{v}\right)p\left(t\right)+\varepsilon \end{array}$$

using the Markov condition with two states *β*_*j*_ = 0; ∀*j* ∈ 1, 2, ⋯, *N* for every *t* ≥ 0, *δ*_*v*_ = *δ*, and *ε*_*j*_ = *ε*, then we can obtain

$$\begin{array}{c} p_{v}\left(t\right)\geq \frac{\varepsilon }{\delta +\varepsilon }. \end{array}$$

In other words, $\frac{\varepsilon }{\delta +\varepsilon }$ is the lower bound for the infection probability when there is no infection rate for every link. Thus, the equation for the upper bound of the infection probabilities is

$$\begin{array}{cc} \frac{dp^{*}\left(t\right)}{dt} & =Adiag\left(\beta_{j}\right)p^{*}\left(t\right)-diag(\frac{\varepsilon }{\delta +\varepsilon })\;Adiag\left(\beta_{j}\right)p^{*}\left(t\right)-\left(\delta +\varepsilon \right)p^{*}\left(t\right)+\varepsilon \\ & =[Adiag\left(\beta_{j}\right)-diag(\frac{\varepsilon }{\delta +\varepsilon })\;Adiag\left(\beta_{j}\right)-\left(\delta +\varepsilon \right)]\;p^{*}\left(t\right)+\varepsilon \\ & =[(I-diag(\frac{\varepsilon }{\delta +\varepsilon }))\;Adiag\left(\beta_{j}\right)-diag\left(\varepsilon +\delta \right)]\;p^{*}\left(t\right)+\varepsilon \\ \frac{dp^{*}\left(t\right)}{dt} & =[diag(\frac{\delta }{\delta +\varepsilon })\;Adiag\left(\beta_{j}\right)-diag\left(\varepsilon +\delta \right)]\;p^{*}\left(t\right)+\varepsilon \end{array}$$ (12)

Let $\bar{Q}=diag(\frac{\delta }{\delta +\varepsilon })\;Adiag\left(\beta_{j}\right)-diag\left(\varepsilon +\delta \right)$ then Eq (12) can be written as

$$\begin{array}{c} p^{{*}^{\prime }}\left(t\right)=\bar{Q}p^{*}\left(t\right)+\varepsilon \end{array}$$ (13)

This equation becomes a nonhomogeneous differential equation that can be solved in the same way as Xu and Hua (2019) [19], and the result is

$$\begin{array}{c} p^{*}\left(t\right)=e^{\bar{Q}t}p^{*}\left(0\right)+{\bar{Q}}^{-1}\left[e^{\bar{Q}t}-I\right]\varepsilon \end{array}$$ (14)

**Proposition 1**. *The upper bound for the stationary infection probability of node v is given by*

$$\begin{array}{c} p_{v\infty }=\frac{\sum_{j=1}^{N}\beta_{j}a_{vj}p_{j\infty }+\varepsilon }{\sum_{j=1}^{N}\beta_{j}a_{vj}p_{j\infty }+\varepsilon +\delta },v=1,\cdots ,N. \end{array}$$ (15)

*where* *p*_*v*∞_ = *lim*_*t*→∞_ *p*_*v*_(*t*).

*Proof*. The dynamics of the upper bound enter a stationary state if ${\text{lim}}_{t\to \infty }p_{v}^{{}^{\prime }}\left(t\right)=0$ for *v* = 1, ⋯, *N*. Consider Eq (9) and lim_*t*→∞_ *p*_*v*_(*t*) = *p*_*v*∞_, we get

$$\begin{array}{cc} \sum_{j=1}^{N}\beta_{j}a_{vj}p_{j\infty }+\varepsilon & =(\sum_{j=1}^{N}\beta_{j}a_{vj}p_{j\infty }+\varepsilon +\delta )p_{v\infty } \end{array}$$ (16)

$$\begin{array}{cc} p_{v\infty } & =\frac{\sum_{j=1}^{N}\beta_{j}a_{vj}p_{j\infty }+\varepsilon }{\sum_{j=1}^{N}\beta_{j}a_{vj}p_{j\infty }+\varepsilon +\delta },v=1,\cdots ,N. \end{array}$$ (17)

### Simulation procedure

We used the simulation procedure provided by Xu and Hua (2019) by modifying the rate of the interarrival time distribution. Let $\bar{\Phi }$ be the set of infected neighbors, where $\bar{\Phi }=\left\{j_{1},j_{2},\cdots ,j_{D_{v}}\right\}\subset \left\{1,2,\cdots ,N\right\}$ and *D*_*v*_ be the number of infected neighbors of node *v*. The time-to-infection of node *v* due to attacks from neighbors is given by the random variables $Y_{j_{1}},Y_{j_{2}},\cdots ,Y_{j_{D_{v}}}$. In the Markov-based model, the random variables have exponential distributions. However, the rate of distribution may differ according to the inhibitory effect of infection at each node. Survival functions with different rates are ${\bar{F}}_{j}\left(x\right)=e^{-\beta_{j}x}$, where *j* ∈ {1, 2, ⋯, *N*} is the index of the node. The time-to-infection due to malicious site access is given by the random variable *Z*_*v*_ with survival function ${\bar{G}}_{v}=e^{-\varepsilon x}$, and the time-to-recovery is an exponential random variable *R*_*v*_ with rate *δ*. Using the theory of alternating renewal processes and the assumption of positive lower orthant dependence [19], the stationary upper bound of infection probability of node *v* is

$$\begin{array}{cc} p_{v\infty } & \leq \frac{E[R_{v}]}{E\left[R_{v}\right]+E_{D_{v}}\left[\int_{0}^{\infty }\prod_{s=1}^{D_{v}}{\bar{F}}_{j_{s}}\left(x\right){\bar{G}}_{v}\left(x\right)dx\right]}\\ & =\frac{\frac{1}{\delta }}{\frac{1}{\delta }+E\left[\int_{0}^{\infty }\prod_{s=1}^{D_{v}}e^{\beta_{j_{s}}x}e^{\varepsilon x}dx\right]}=\frac{\frac{1}{\delta }}{\frac{1}{\delta }+E\left[\int_{0}^{\infty }e^{\sum_{s=1}^{D_{v}}\beta_{j_{s}}x}e^{\varepsilon x}dx\right]} \end{array}$$ (18)

Consider that $\sum_{s=1}^{D_{v}}\beta_{j_{s}}=\sum_{j=1}^{N}\beta_{j}a_{vj}I_{j}$, using Jansen’s inequality Eq (18) can be written as

$$\begin{array}{cc} p_{v\infty } & \leq \frac{\frac{1}{\delta }}{\frac{1}{\delta }+\int_{0}^{\infty }e^{E[\sum_{j=1}^{N}\beta_{j}a_{vj}I_{j}]x}e^{\varepsilon x}dx]}=\frac{\frac{1}{\delta }}{\frac{1}{\delta }+\int_{0}^{\infty }e^{(\sum_{j=1}^{N}\beta_{j}a_{vj}p_{j}+\varepsilon )x}dx}\\ & =\frac{\sum_{j=1}^{N}\beta_{j}a_{vj}p_{j\infty }+\varepsilon }{\sum_{j=1}^{N}\beta_{j}a_{vj}p_{j\infty }+\varepsilon +\delta },v=1,\cdots ,N. \end{array}$$ (19)

The result in Eq (19) is a stationary upper bound, which is the same as the result of the IH-SIS model in Proposition 1. Thus, the simulation can be carried out using the procedure given by Algorithm 1.

**Algorithm 1**: Simulation of cybersecurity risk with clustering coefficient factor.

**Input**: Local clustering coefficient of node *C*_*v*_, basic infection rate *β*, initial status, the number of simulations *n*_*sim*_, contract period *T*, set of susceptible nodes.

Calculate the infection rate with inhibiting factor *β*_*v*_ = *βf*(*C*_*v*_), *v* = 1, ⋯, *N*.

 **for** *i* = 1 **to** *n*_*sim*_ **do**

 **while** *t* < *T* **do**

  Calculate the number of infected nodes $\tilde{M}$.

  Generate random time-to-recovery $r_{1},r_{2},\cdots ,r_{\tilde{M}}$ from *exp*(*δ*).

  **for** *v* **in** *secure nodes* **do**

   Determine the infected neighbors of node *v*, $j_{1},\cdots ,j_{d_{v}}$.

   Generate random time-to-infection $y_{j_{1}},y_{j_{2}},\cdots ,y_{j_{d_{v}}}$ based on their infection rate from *exp*(*β*_*j*_), *j* ∈ 1, 2, ⋯, *N*.

   Generate time-of-self-infection *z*_*v*_ from *exp*(*ε*).

  **end**

  Determine time for the first event $t_{1}=min\left\{r_{1},r_{2},\cdots ,r_{\tilde{M}},y_{v_{j_{1}}},y_{v_{j_{2}}},\cdots ,y_{v_{j_{d_{v}}}},z_{v}\right\}$.

  **if** *infection occurs* **then**

   Change status from 0 to 1 and calculate the loss.

  **else**

   Change status from 0 to 1 and calculate the loss.

  **end**

 **end**

 **return** *t*, *network status, the loss for every node*

**end**

Calculate insurance premium until *T*.

**Output**: network status, total loss, premiums.

## Results and discussion

In this section, we discuss the results of the theory and simulations that have been carried out. The simulation was carried out for the contract time *T* = 100 days. The selected input parameters were *β* = 0.2, *δ* = 1, and *ε* = 0.2. To analyze the inhibitory effect, other parameters were set the same, including the degree of the node. Therefore, the study was carried out on the regular network and its properties. A regular graph was generated for the orders *n* = 20 and *k* = 4. For the loss function, *L*_*v*_ followed the Beta distribution with density function

$$\begin{array}{c} f_{L_{v}}\left(\phi |a,b,c,{\tilde{w}}_{v}\right)=\frac{c}{\phi B(a,b)}{\left(\frac{\phi }{{\tilde{w}}_{v}}\right)}^{ac}{\left(1-{\left(\frac{\phi }{{\tilde{w}}_{v}}\right)}^{c}\right)}^{b-1},0<\phi <{\tilde{w}}_{v}, \end{array}$$ (20)

where ${\tilde{w}}_{v}$ is the scale parameter used to describe the wealth of node or device *v*, *a*, *b*, *c* > 0 are shape parameters, and *B* is the beta function. We chose *a* = 3, *b* = 8, *c* = 1, and ${\tilde{w}}_{v}=1500$ for this case. The cost function for infection-related loss and system downtime-related loss is described as

$$\begin{array}{c} \mu_{v}\left(L_{v}=\phi \right)=\psi \phi ,\;\xi_{v}\left(R_{v}=r_{v}\right)=\psi_{1}{\tilde{w}}_{v}+\psi_{2}r_{v} \end{array}$$ (21)

where *ψ*, *ψ*_1_, *ψ*_2_ are rates related to infection, initial wealth, and recovery process. The cost function parameter was chosen so that (*ψ*, *ψ*_1_, *ψ*_2_) = (1 × 10^−3^, 5 × 10^−6^, 2 × 10^−5^). The premium until time *t* is calculated using the standard deviation principle [42] as follows:

$$\begin{array}{c} P\left(t\right)=E\left[S\left(t\right)\right]+\xi \sqrt{Var(S(t))} \end{array}$$ (22)

where the loading factor *ξ* = 0.15.

**Fig 4:**
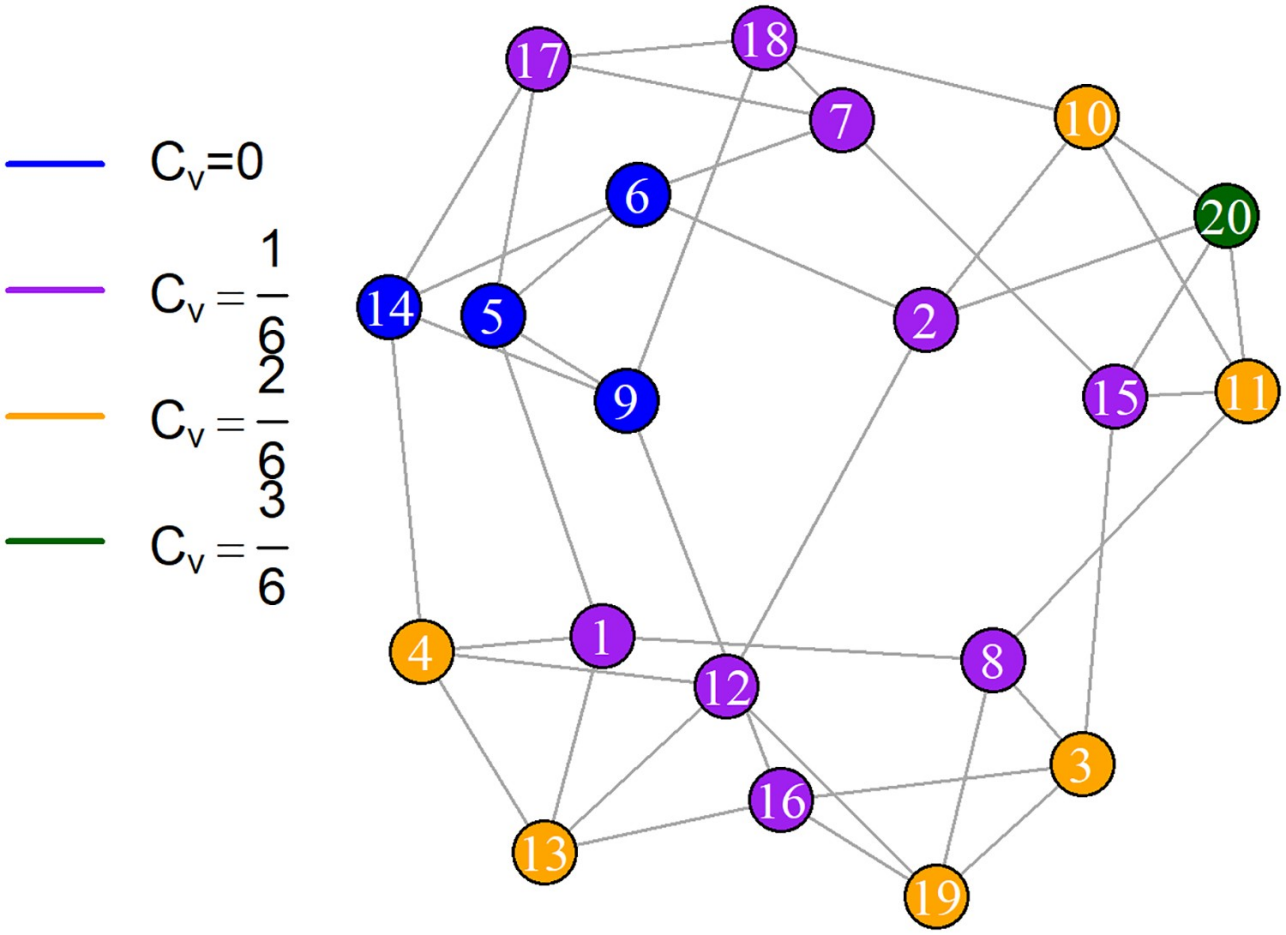
Study case in 4-regular graph. Realization of a random 4-regular graph with the order *n* = 20.

A discussion of these results, including the theory and simulation of premiums, is obtained on a *k*-regular graph. Numerical studies were conducted on the 4-regular graph provided by Fig 4.

### Clustering coefficient in *k*-regular graph

**Fig 5:**
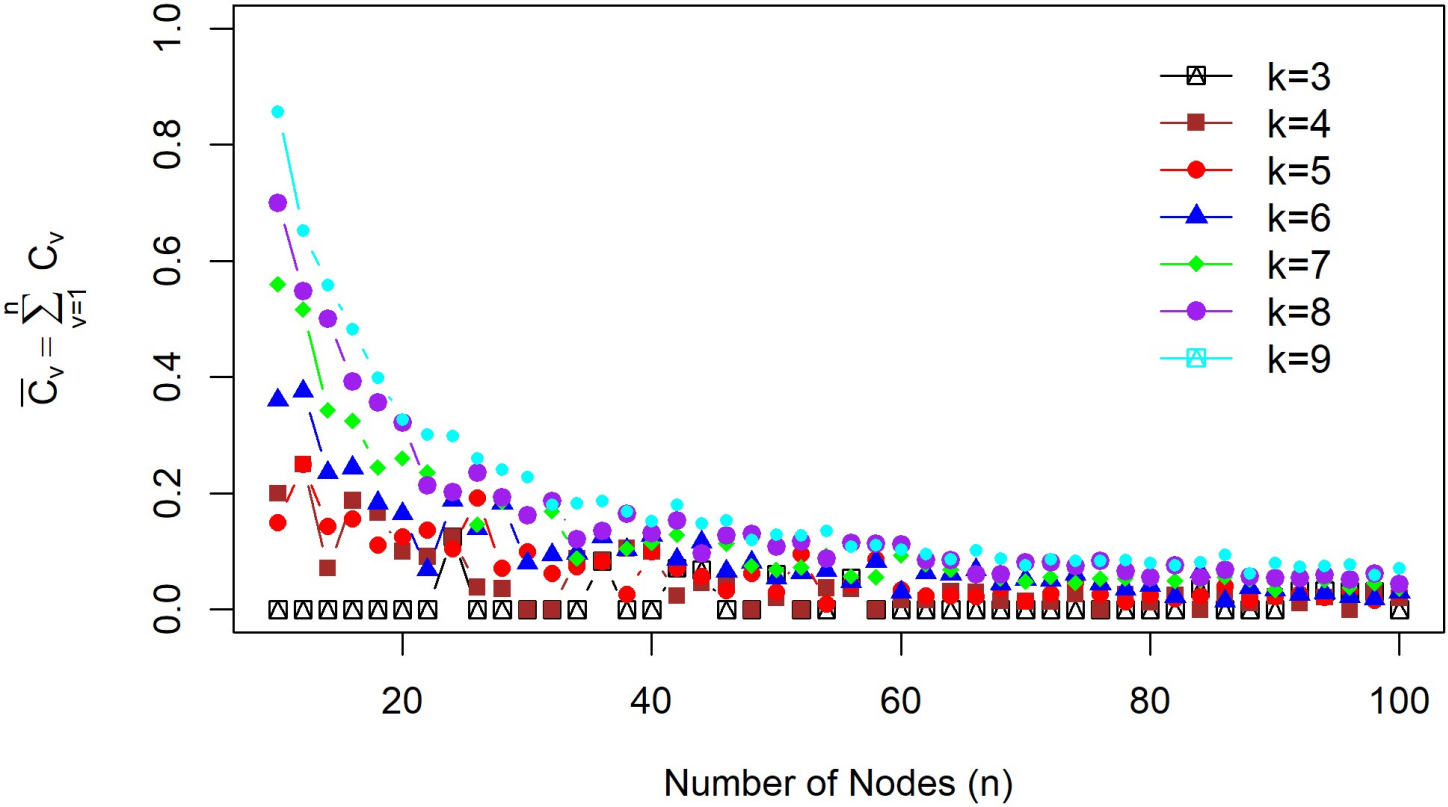
Average local clustering coefficient. Relationship between the average clustering coefficient $\frac{1}{n}\sum_{v=1}^{n}C_{v}$ and the order of graph *n* = 10, 12, 14, ⋯, 100 for several different *k* = 1, 2, ⋯, 9.

The relationship between the clustering coefficient and the order of the regular graph is given by Fig 5. The average of the local clustering coefficients grows as the degree of a node *k* increases for each *n*. This result shows the average clustering coefficient that approaches 0 as the *n* order becomes more extensive. Thus, if *n* is very large and *k* is very small, it can be concluded that there is a minimal clustering coefficient effect on the pricing procedure on the *k*-regular graph.

Some of the theoretical results obtained concerning the clustering coefficient and premium calculation are as follows.

**Lemma 4** (Minimum effect). *For* 2—*connected regular graph G* = (*V*, *E*) *with* *n* > 3, *the clustering coefficient for each node is zero. In this case, there are minimum effects of the clustering coefficient on cyber insurance premiums* ∀*v* ∈ *V*.

*Proof*. All 2-connected regular graphs for *n* > 3 are cycle graphs (ring networks). Thus, for all {*u*, *v*, *w*} ⊂ *V*, no triangles are formed, so (*u*, *v*), (*u*, *w*) ∈ *E* but (*v*, *u*) ∉ *E*. The implication is *T*(*v*) = 0 and *C*_*v*_ = 0, ∀*v* ∈ *V*. Consider the conditions for the cluster function *f*(*C*_*v*_), namely, $\frac{df(C_{v})}{dC_{v}}$ and 0 < *f*(*C*_*v*_) < 1. Additionally, consider the effect of the clustering coefficient on the spread of the epidemic as *β*_*v*_ = *βf*(*C*_*v*_). Because the *f*(*C*_*v*_) function decreases, when *C*_*v*_ is at its minimum value, *f*(*C*_*v*_) is at its maximum value; in other words, *βf*(*C*_*v*_) → *β* for *f*(*C*_*v*_) → 1, and there is a minimum decreasing effect of the clustering coefficient on the spread of the epidemic and the pricing of cyber insurance premiums.

**Lemma 5** (Maximum effect). *For a* (*n* − 1)-*connected regular graph* *G*(*V*, *E*) *with* *n* ≥ 3, *the clustering coefficient for each node is one. In this case, there are maximum effects of the clustering coefficient on the pricing of cyber insurance premiums* ∀*v* ∈ *V*.

*Proof*. All (*n* − 1)-regular graphs for *n* ≥ 3 are complete graphs (*K*_*n*_). Thus, for all {*u*, *v*, *w*} ⊂ *V*, triangles are always formed so that (*u*, *v*), (*u*, *w*), (*v*, *u*) ∈ *E*, ∀*u*, *v*, *w* ∈ *V*. The implications are $T\left(v\right)=\frac{\left(n-1)(n-2\right)}{2}$ and *C*_*v*_ = 1, ∀*v* ∈ *V*. Consider the conditions for the *f*(*C*_*v*_) clustering function, namely, $\frac{df(C_{v})}{dC_{v}}$ and 0 < *f*(*C*_*v*_) < 1. Additionally, consider the effect of the clustering coefficient on the spread of the epidemic as *β*_*v*_ = *βf*(*C*_*v*_). Because *f*(*C*_*v*_) is a decreasing function, when *C*_*v*_ is at its maximum value, *f*(*C*_*v*_) is at its minimum value, in other words, *βf*(*C*_*v*_) → *min*{*β*_*j*_} for *f*(*C*_*v*_) → *min*{*f*(*C*_*v*_)}. Thus, there are maximum decreasing effects of the clustering coefficient on the spread of the epidemic and the pricing of cyber insurance premiums.

The last two lemmas bring us to the following consequences:

**Corollary 2**. *There is a minimum of one or more structures on a k-connected regular graph for k* = 3, ⋯, *n* − 2 *such that there is at least one node that has nonzero and not one clustering coefficient. Thus, there is an effect on a node in cyber insurance rate making with*

$$\begin{array}{c} min\left\{f\left(C_{v}\right)\right\}<f\left(C_{v}\right)<maks\left\{f\left(C_{v}\right)\right\}\Leftrightarrow min\left\{\beta_{v}\right\}<\beta {}_{v}<maks\left\{\beta_{v}\right\} \end{array}$$ (23)

*Proof*. Based on the results of Lemma 4 and Lemma 5, there is always a structure of *k*-regular graph for *k* = 3, ⋯, *n* − 2 with the specified order *n* and holds the existence of a regular graph that is *nk* even. This is because the formation process of the *k*-regular graph for *k* = 3, ⋯, *n* − 2 involves adding one link to the 2-connected regular graph or subtracting one link at the *n* − 1-connected regular graph continuously. As a consequence, at least one node in that structure with 0<T(v)<(kv2) indicates that 0 < *C*_*v*_ < 1. Thus applies

$$\begin{array}{c} min\left\{f\left(C_{v}\right)\right\}<f\left(C_{v}\right)<maks\left\{f\left(C_{v}\right)\right\}\Leftrightarrow min\left\{\beta_{v}\right\}<\beta {}_{v}<maks\left\{\beta_{v}\right\} \end{array}$$ (24)

The three functions explaining the inhibitory effect of the clustering coefficient are defined as follows:

- The linear function is *f*(*C*_*v*_) = −*C*_*v*_ + 1.
- The quadratic function is $f\left(C_{v}\right)=-0.65C_{v}^{2}+1$ [33].
- The exponential function is $f\left(C_{v}\right)=-\sqrt{C_{v}}+1$.

Each function provides a different inhibitory effect. The choice of the operation depends on how much the community can reduce the effectiveness of the infection rate. The quadratic function represents low inhibition, the linear function represents moderate inhibition, and the exponential function represents high inhibition. The numerical studies in the following subsection consider these three functions.

### Upper bound of infection probability

**Fig 6:**
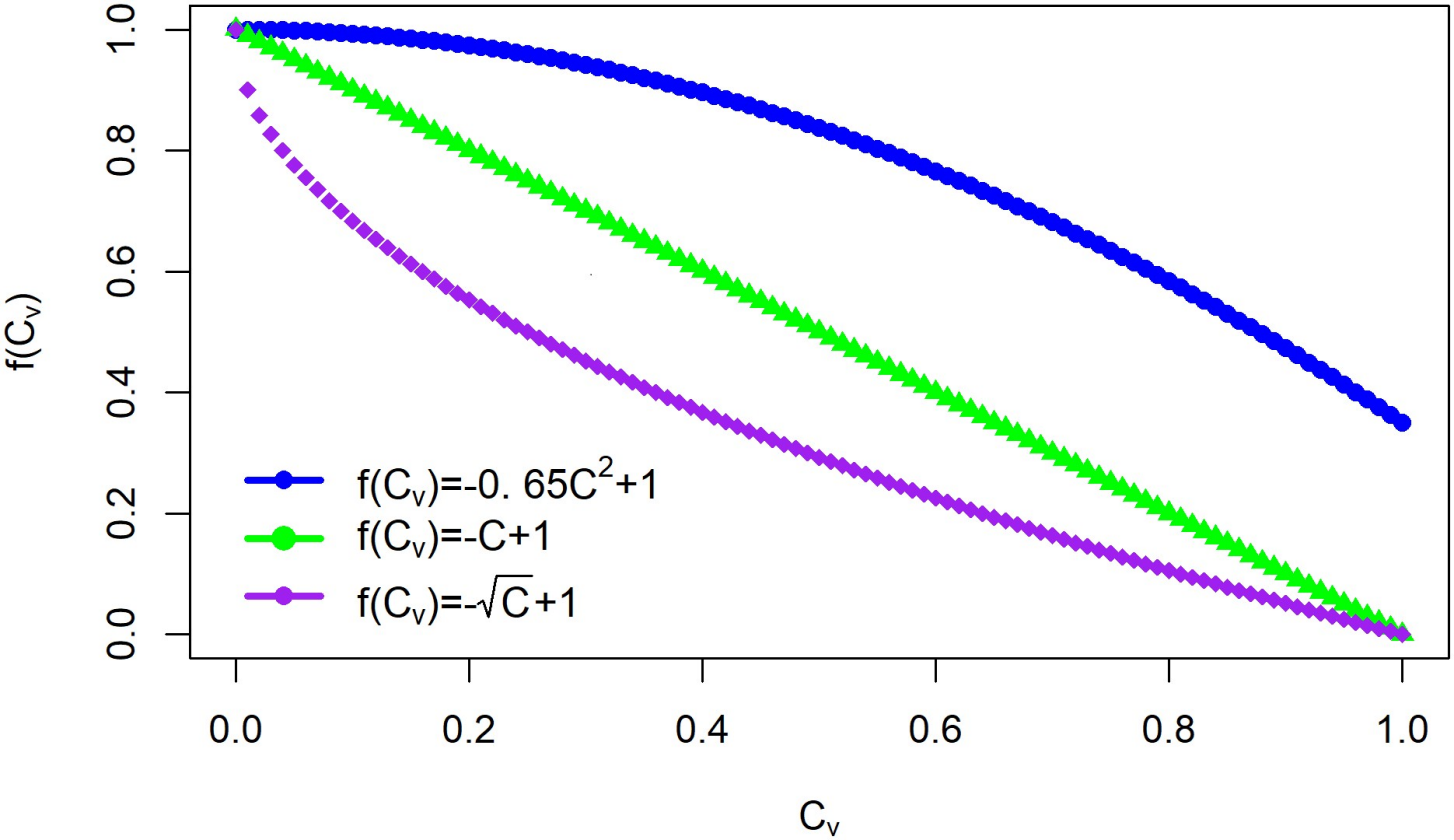
Representation of the inhibition of the epidemic by the clustering coefficient. The inhibition considers three functions, namely, linear (*f*(*C*_*v*_) = −*C*_*v*_ + 1), quadratic ($f\left(C_{v}\right)=-0.65C_{v}^{2}+1$), and exponential ($f\left(C_{v}\right)=-\sqrt{C_{v}}+1$) functions.

**Fig 7:**
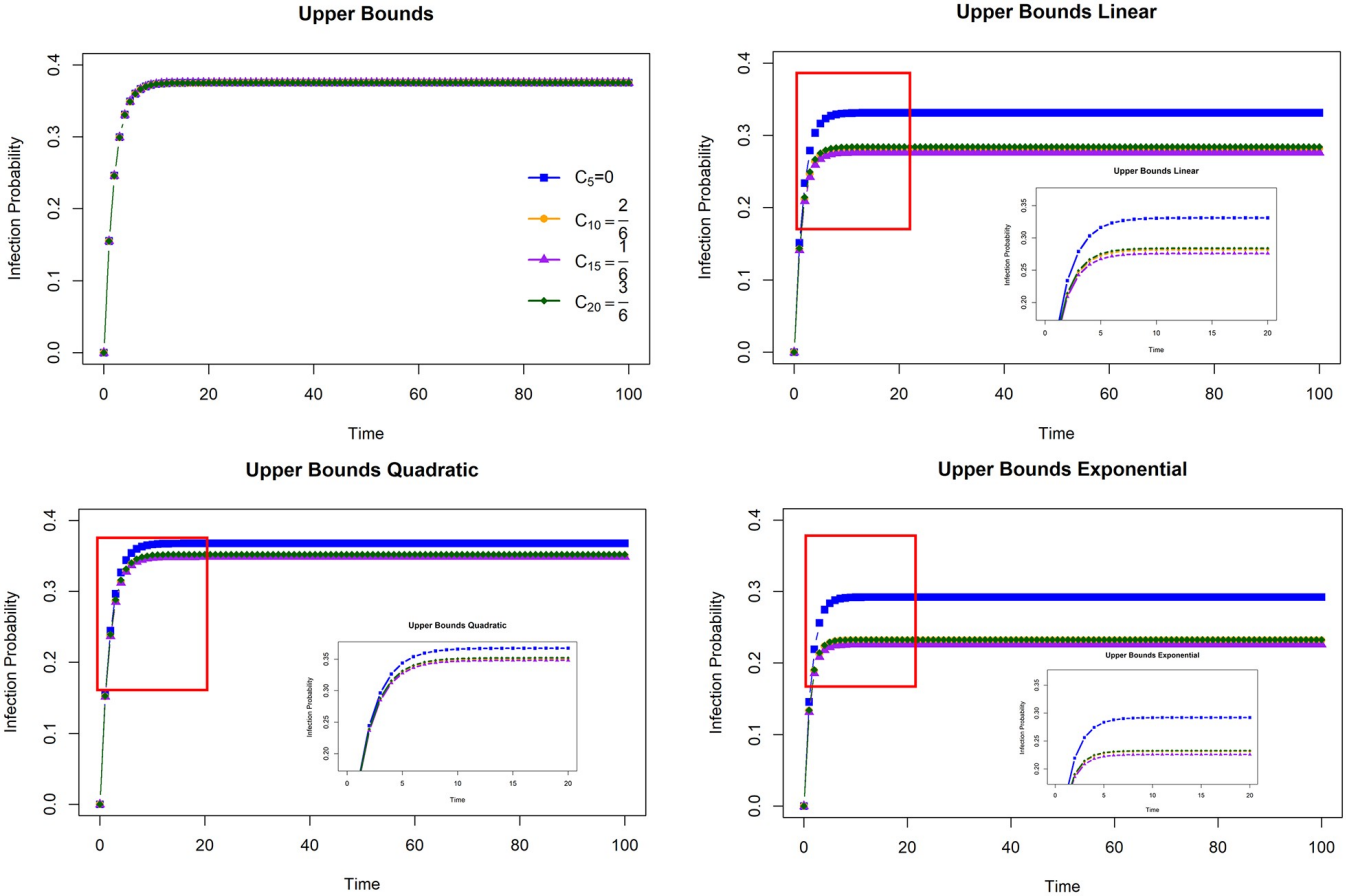
The upper bounds for infection probabilities. A comparison between upper bounds for infection probabilities without and with clustering coefficients using three types of inhibition functions (linear, quadratic, and exponential). The red box reflects the upper bound for resizing all figures at *t* = [3,20], and the extension outcomes can be seen inside each figure.

The upper bound of infection probabilities was obtained from Eq (12) in Theorem 1. The three functions in Fig 6 demonstrate the influence of the magnitude of the inhibition on the upper bound. We compared the upper bound with and without inhibitory effects. Fig 7 shows the upper bound of infection probabilities for four nodes, namely, node 5, node 10, node 15, and node 20. Each node represents a different clustering coefficient. The clustering coefficients of nodes 5, 10, 15, and 20 are zero, $\frac{1}{6}$, $\frac{2}{6}$, and $\frac{3}{6}$, respectively.

The effect of structural characterization with local clustering coefficients is visible. The upper bound obtained by the model without the clustering coefficient is the same as that obtained by Xu and Hua (2019) [19]. It can be seen from the upper bound of each node that coincides with each other. By studying regular graphs, Antonio and Indratno (2021) [26] support the substantial effect of degrees on the model. Other factors that impact the rate of infection have not been explored in this model. However, the clustering structure can affect the speed or effectiveness of propagation, where nodes can have different infection rates [32, 33]. Through local clustering coefficients, each node undergoes an infection rate adjustment that depends on the inhibitory function. The three inhibiting parts considered earlier had different impacts on the upper bound of infection probabilities. The upper bound with the quadratic function gives a slight change compared to the upper bound without the clustering coefficient effect. Then, the linear function has a moderate impact, and the exponential function has a reasonably strong influence. Therefore, these functions represent the level of impact of the clustering structure on the speed and effectiveness of the spread of the virus.

Based on the model in Eq (7), the transition probability of a node depends on the sum of the clustering coefficient functions of its neighbors. The upper bounds of the three functions have the same pattern. Node 5 always produces the highest upper bound and is followed by nodes 20, 10, and 15. Table 1 summarizes the clustering coefficients of the four neighbors of each node, the total clustering coefficients and the totals of the three functions. This fact supports the upper bound result. Node 5 produces the highest total clustering coefficient functions for linear, quadratic, and exponential functions. Sequentially, nodes 20, 10, and 15 have a total clustering coefficient in the linear, quadratic and exponential functions below node 5. This confirms that the upper bound depends on the clustering coefficient function of the neighbors.

**Table 1:** Characteristics of clustering coefficients for nodes in a 4-regular graph topology (Fig 4).

Nodes	Clustering Coefficient of Neighbors				Total	Quadratic	Linear	Exponential
1	0.3333	0.0000	0.1667	0.3333	0.8333	3.8375	3.1667	2.4371
2	0.0000	0.3333	0.1667	0.5000	1.0000	3.7472	3.0000	2.3073
3	0.1667	0.1667	0.1667	0.3333	0.8333	3.8736	3.1667	2.1979
4	0.1667	0.1667	0.3333	0.0000	0.6667	3.8917	3.3333	2.6062
**5**	**0.1667**	**0.0000**	**0.0000**	**0.1667**	**0.3333**	**3.9639**	**3.6667**	**3.1835**
6	0.1667	0.0000	0.1667	0.0000	0.3333	3.9639	3.6667	3.1835
7	0.0000	0.1667	0.1667	0.1667	0.5000	3.9458	3.5000	2.7753
8	0.1667	0.3333	0.3333	0.3333	1.1667	3.7653	2.8333	1.8597
9	0.0000	0.0000	0.1667	0.1667	0.3333	3.9639	3.6667	3.1835
**10**	**0.1667**	**0.3333**	**0.1667**	**0.5000**	**1.1667**	**3.7292**	**2.8333**	**1.8990**
11	0.1667	0.3333	0.1667	0.5000	1.1667	3.7292	2.8333	1.8990
12	0.1667	0.3333	0.3333	0.3333	1.1667	3.7653	2.8333	1.8597
13	0.1667	0.3333	0.1667	0.1667	0.8333	3.8736	3.1667	2.1979
14	0.3333	0.0000	0.0000	0.1667	0.5000	3.9097	3.5000	3.0144
**15**	**0.3333**	**0.1667**	**0.3333**	**0.5000**	**1.3333**	**3.6750**	**2.6667**	**1.7299**
16	0.3333	0.0000	0.3333	0.3333	1.0000	3.7833	3.0000	2.2679
17	0.0000	0.1667	0.0000	0.1667	0.3333	3.9639	3.6667	3.1835
18	0.1667	0.0000	0.3333	0.1667	0.6667	3.8917	3.3333	2.6062
19	0.3333	0.1667	0.1667	0.1667	0.8333	3.8736	3.1667	2.1979
**20**	**0.1667**	**0.3333**	**0.3333**	**0.1667**	**1.0000**	**3.8194**	**3.0000**	**2.0288**

Local clustering coefficient of neighbors, total local clustering coefficient, and total inhibition function with linear (*f*(*C*_*v*_) = −*C*_*v*_ + 1), quadratic ($f\left(C_{v}\right)=-0.65C_{v}^{2}+1$), and exponential ($f\left(C_{v}\right)=-\sqrt{C_{v}}+1$).

**Fig 8:**
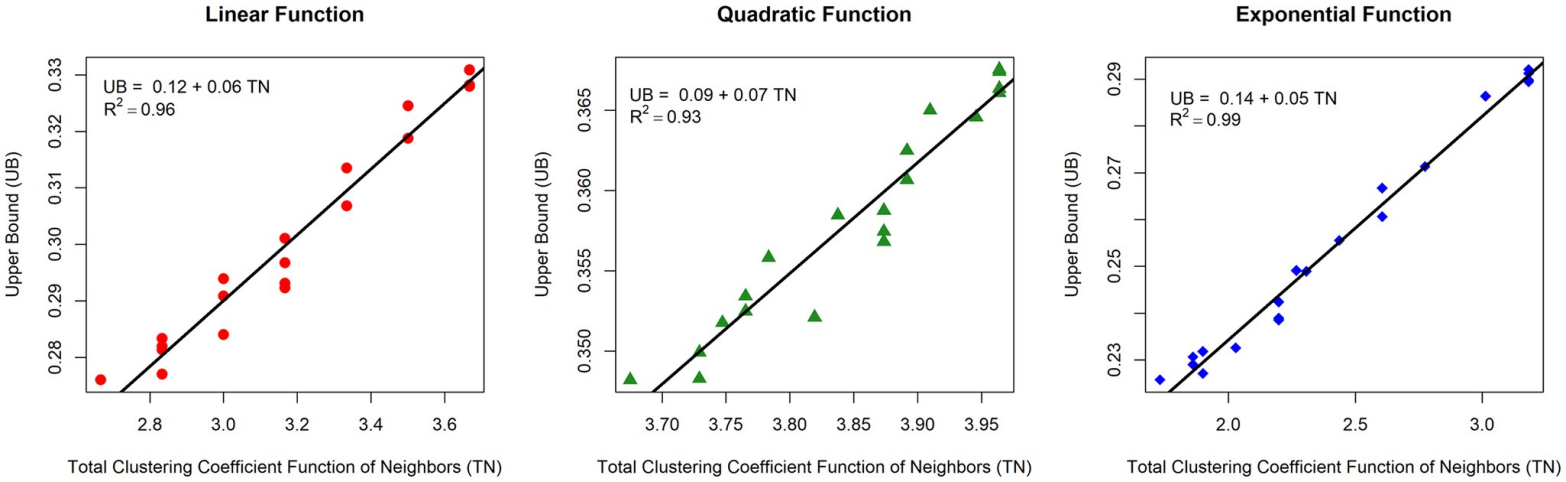
TN and UB relationship of twenty nodes in a 4-regular graph on linear, quadratic, and exponential functions.

We looked at the linear relationship between the total clustering coefficient function (TN) and the upper bound (UB) to prove this assertion. The outcome is depicted in Fig 8. For all three functions, the figure depicts a positive linear relationship between TN and UB, which means that while TN grows, UB grows as well. The linear relationship is given by

$$\begin{array}{c} \text{UB}=\alpha_{0}+\alpha_{1}\text{TN}. \end{array}$$

*α*_0_ represents the intercept, *α*_1_ represents the slope of the linear model, and *R*^2^ represents the coefficient of determination. The coefficient of determination measures how well the independent variable can predict the fluctuation of the dependent variable. The linear relationship is powerful when *R*^2^ is close to one. When *R*^2^ is close to one, the linear connection is quite strong. Let $R_{L}^{2},R_{Q}^{2}$, and $R_{E}^{2}$ be *R*^2^ for linear, quadratic, and exponential functions. With $R_{L}^{2},R_{Q}^{2},R_{E}^{2}>0.9$, the three functions have a strong relationship. As a result, TN can account for more than 90% of UB. For linear, quadratic, and exponential functions, *α*_0_ is 0.12, 0.09, and 0.14, respectively. For linear, quadratic, and exponential functions, *α*_1_ is 0.06, 0.07, and 0.05, respectively. The upper bound is affected more strongly by the exponential inhibition function. As a result, it is obvious that the risk of transmission is no longer homogenous (same upper bound when degrees are equal) but instead has a significant correlation with the total inhibitory function of neighbors.

### Premiums setting

**Fig 9:**
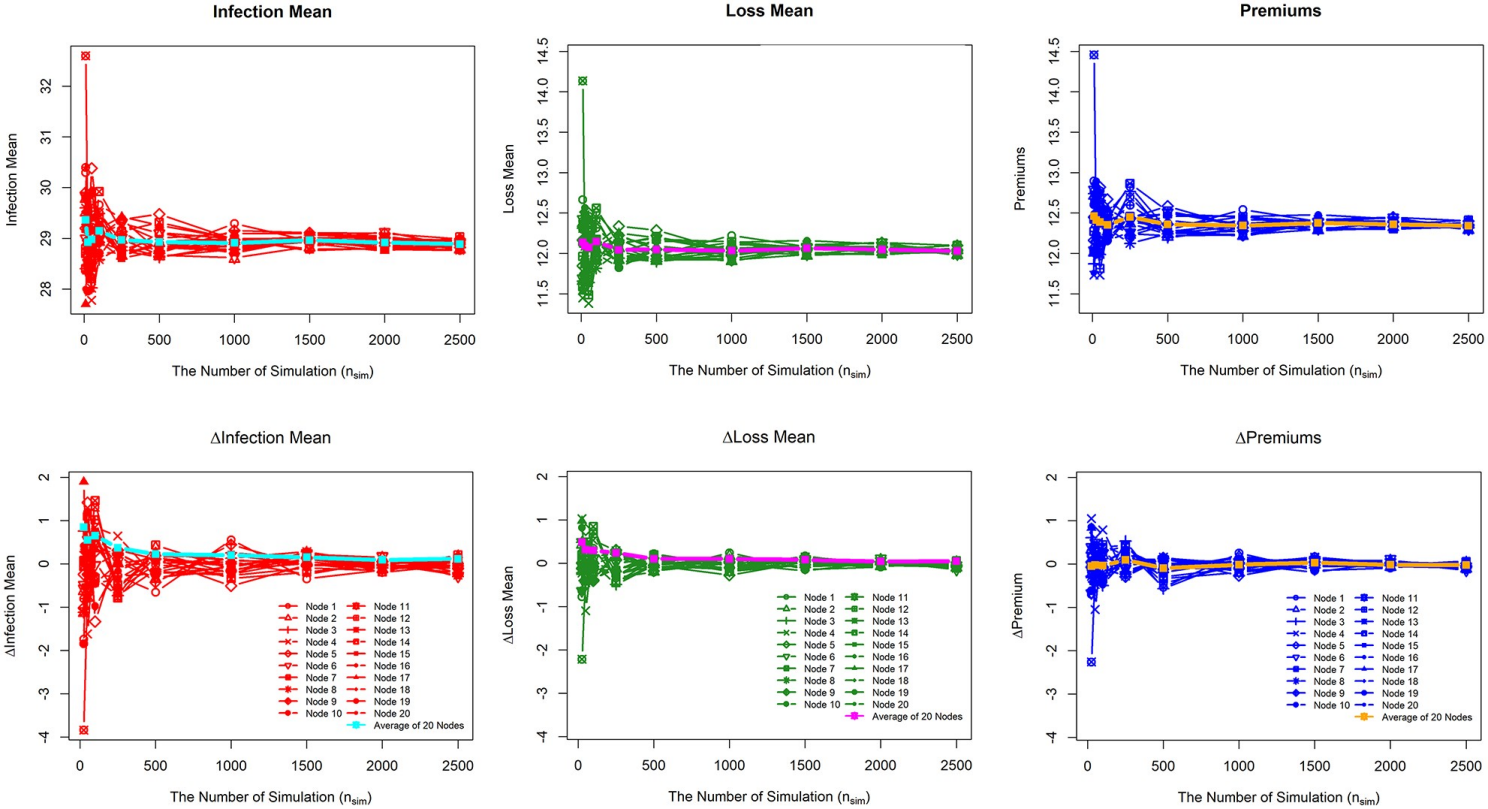
Convergence of the Monte Carlo simulation. Convergence for Infection Mean, Loss Mean, and Premiums.

We performed simulations using Algorithm 1 to produce premiums. Cyber incident data are generated based on transmission parameters. Determining the number of simulations (*n*_*sim*_) is one of the challenges of this method. We considered ten numbers of simulations. *n*_*sim*_ = {10, 25, 50, 100, 250, 500, 1000, 1500, 2000, and 2500} to find the convergence of *n*_*sim*_. We ran simulations with *β* = 0.2 and no inhibition from local clustering coefficients to demonstrate convergence. Fig 9 reveals the convergence of the Monte Carlo simulation for mean infection, mean loss, and premiums of 20 nodes. For each variable, the average of 20 nodes is also displayed. In addition, the difference (Δ) for each (*n*_*sim*_) is taken into consideration. When *n*_*sim*_ is increased, all three variables converge to the same value. At *n*_*sim*_ = 500, all figures are convergent on average. However, divergence is still apparent for each node at *n*_*sim*_ = 500. At *n*_*sim*_ = 2000, each node has begun to converge. If the difference (Δ) between the number of simulations is close to or equal to zero, the percentage change (Δ) implies convergence. As seen in Fig 9 for the variables ΔInfection Mean, ΔLoss Mean, and ΔPremiums, all nodes and their averages approach zero as *n*_*sim*_ is increased, and the simulation is considered to be convergent at *n*_*sim*_ = 2000. Finally, for the premium set, we choose *n*_*sim*_ = 2000 as the number of simulations.

**Fig 10:**
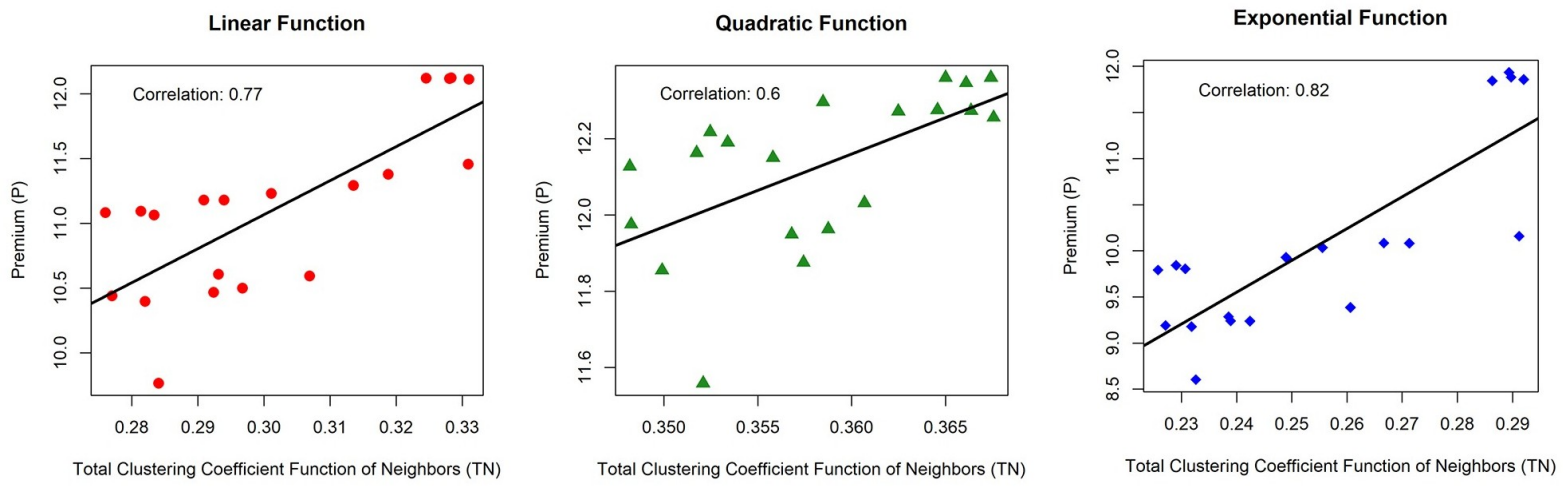
Correlation between the total function of the clustering coefficient (TN) and the premium (P). TN and P relationship of twenty nodes in a 4-regular graph on linear, quadratic, and exponential functions.

On the 4-regular graph, premiums have been modified to account for the clustering structure. The linear relationship (correlation) between the total linear, quadratic, and exponential inhibitory functions (TN) and the premium is visualized in Fig 10. For twenty nodes with linear, quadratic, and exponential functions, the correlation between TN and P is more than 0.6, suggesting a strong and moderately strong linear relationship. The correlations for the linear, quadratic, and exponential functions are 0.77, 0.66, and 0.82, respectively.

TN is a representation of two network entities: the degree and the local clustering coefficient. As a result, these findings incorporate the influence of the clustering structure on the premium. If the premium is based just on degrees, it is often homogenous. Indeed, the clustering structure influences the efficacy of epidemic propagation. This fact shows that when the effect of the inhibitory function increases or the speed of epidemic spread decreases due to the clustering coefficient, then the premium corresponds to the total inhibitory function of the neighbors. Additionally, these findings suggest the existence of a significant linear connection between UB and TN. UB has been verified as the initial premium estimate.

The premiums for the twenty nodes on the four-regular graph are shown in Table 2. Nodes 5, 10, 15, and 20 were bolded to illustrate the presence of various local clustering coefficients. The overall inhibitory function (TN) adjusted premiums in line with TN. As in the upper bound (UB), this outcome is impacted significantly by the TN in Table 1. Premiums without clustering coefficients (without CC) are compared, along with linear, quadratic, and exponential inhibition functions. The premiums without CC in Xu and Hua’s (2019) model [19] are 12.3 units. Each node has four degrees in total. As previously stated, TN accounts for two network properties: degree and the local clustering coefficient. TN has a premium of approximately 12.3 units while having a value close to 4. Conversely, the TN that is less than the degree corrects the premiums by the difference between TN and degrees. Node 5, with the largest TN for linear, quadratic, and exponential functions, provides the most extensive premium in comparison to other nodes. TN decreases when the trend of inhibitory function decreases, resulting in a decrease in the premium reduction trend. Premiums with an exponential inhibition function are the least expensive option. The premiums are more realistic than when only degrees are included. Additionally, the premium is not uniform but is adapted according to the cluster structure of its neighbors. Premiums that use this strategy might be cheaper, making them more competitive in the market.

**Table 2:** Premiums in a 4-regular graph topology (Fig 4).

Node	Without Clustering Coefficient	With Clustering Coefficient		
		Quadratic	Linear	Exponential
1	12.308	12.2962	11.2318	10.0359
2	12.3227	12.1626	11.1794	9.9281
3	12.3873	11.9489	10.4696	9.2865
4	12.3309	12.0312	10.5942	9.3866
**5**	**12.395**	**12.3603**	**12.1119**	**11.858**
6	12.4456	12.3462	12.1239	11.8813
7	12.3034	12.2755	11.3785	10.0821
8	12.3825	12.2177	11.0952	9.8431
9	12.4488	12.2736	12.1188	11.9349
**10**	**12.3156**	**11.8545**	**10.3977**	**9.1788**
11	12.3853	11.9751	10.4408	9.1903
12	12.3594	12.1902	11.0635	9.8051
13	12.4054	11.9629	10.5008	9.2387
14	12.3593	12.36	12.1212	11.8433
**15**	**12.318**	**12.1271**	**11.0843**	**9.791**
16	12.4239	12.1504	11.1812	9.9181
17	12.2944	12.2557	11.457	10.1586
18	12.3281	12.2719	11.2924	10.0855
19	12.3437	11.8751	10.6086	9.2408
**20**	**12.3384**	**11.5583**	**9.7662**	**8.6027**
Total	247.1958	242.4932	222.2169	201.2892

Premium without clustering coefficient effect, and with clustering coefficient effect using linear (*f*(*C*_*v*_) = −*C*_*v*_ + 1), quadratic ($f\left(C_{v}\right)=-0.65C_{v}^{2}+1$), and exponential ($f\left(C_{v}\right)=-\sqrt{C_{v}}+1$) inhibition functions.

### Application on real network

**Fig 11:**
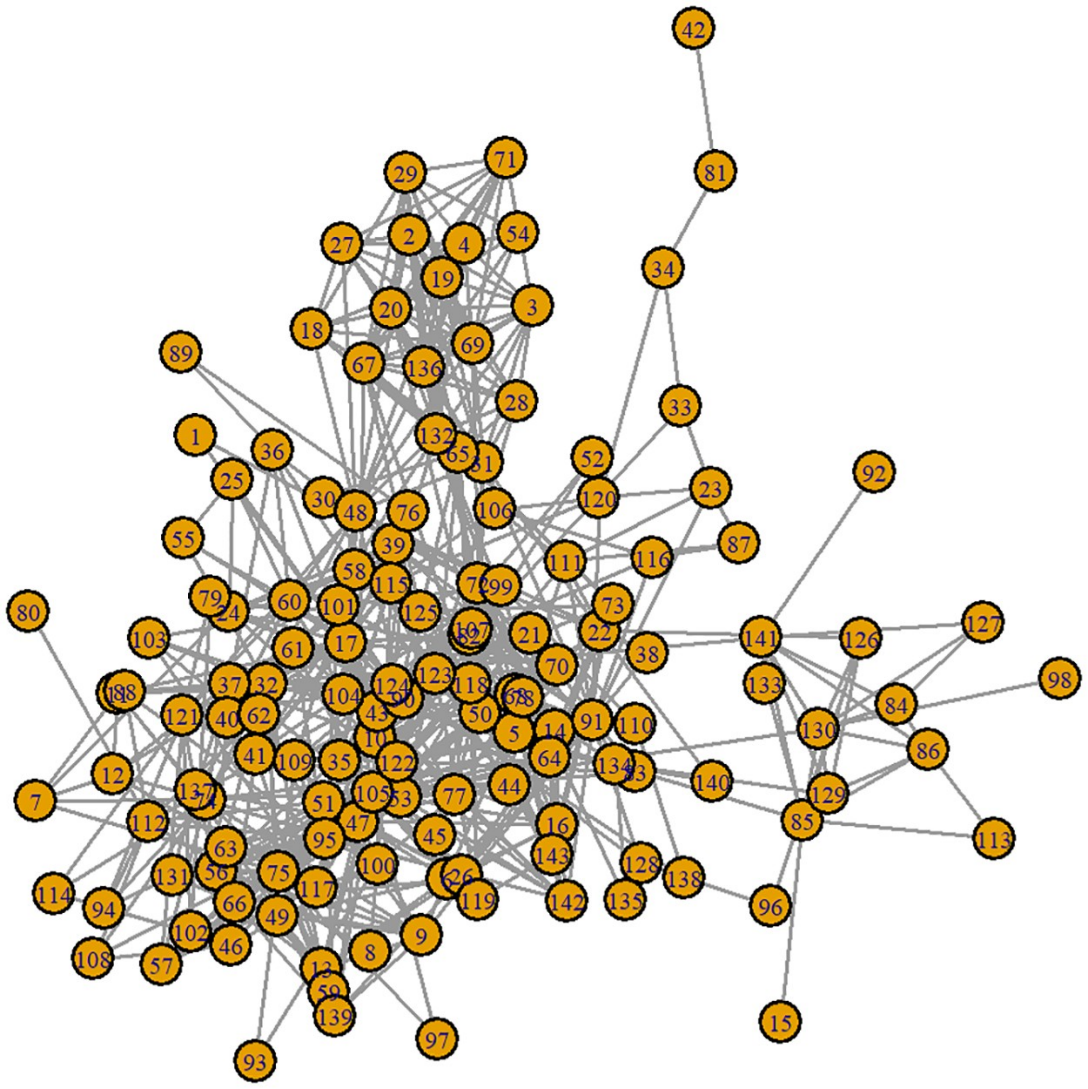
Real communication network. An email-Enron network with nodes representing email accounts or devices and links representing email exchange.

To validate the results, we used a real communication network (see Fig 11). The real network is an email communication network. Rossi and Ahmed (2015) [43] provided communication data, which may be viewed online (https://networkrepository.com/email-enron-only.php). Table 3 presents the characteristics of the email-Enron network in the form of the number of nodes (|*V*|), number of links (|*E*|), density (D), maximum degree (*d*_*max*_), minimum degree (*d*_*min*_), the mean of degrees (*d*_*avg*_), the number of triangles (|*T*|), average triangles formed by a link (|*T*|_*avg*_), the maximum number of triangles formed by a link (|*T*|_*max*_), the average clustering coefficient (*C*_*avg*_), and the global clustering coefficient (*C*).

**Table 3:** Characteristics of an email-Enron network.

Characteristic	|*V*|	|*E*|	*D*	*d*_*max*_	*d*_*min*_	*d*_*avg*_	|*T*|	|*T*|_*avg*_	|*T*|_*max*_	*C*_*avg*_	*C*
Value	143	623	0.0613	42	1	8	2700	18	125	0.4339	0.3590

According to these parameters, this network has 143 nodes and 623 links. With the density *D* equal to 0.0613, this network is classified as of extremely low density. Out of the 142 potential communications, the maximum communication (*d*_*max*_) occurs only between 42 accounts (neighbors). *C*_*avg*_ and *C* can be used to characterize the clustering structure of this network. *C*_*avg*_ = 0.4339 shows that some nodes have a high local clustering coefficient, while others have a low coefficient. The clustering coefficient at the global level is *C* = 0.3590. This measure suggests that |*T*| = 2700 accounts for approximately 35.9% of all triangles constructed in this network. If we focus just on the degree component, we see that nodes with a high degree have an increased risk and premium. However, the more neighbors a node has, the less successful it is in spreading the disease. By including the clustering structure of neighbors in the model, the premium for nodes of the same degree may be rendered in-homogeneous.

On large networks, the simulation complexity increases significantly and takes ample time. Because of these conditions, we modified the transmission parameters in the simulation of a real network. We dropped the infection rate to *ε* = 0.05 and boosted the recovery rate to *δ* = 10 for each node. The modification implies that the average time to infection of a device due to clicking on malicious emails has grown to 20 days. The average time-to-recovery of a device has increased to 2.4 hours. Parameters are chosen based on the assumption that the security system for each device is more robust and the ability to recover is faster in a large company. We compare the computations with and without the inhibitory function to demonstrate the influence on premiums. *n*_*sim*_ = 2000 was used in the calculations to account for simulation convergence.

We chose ten users from a total of 143 to highlight the significance of the findings. The node is selected based on its degree, the overall clustering coefficient of its neighbors, and its location. Table 4 summarizes the ten nodes chosen, along with the parameters that impact the premium. The nodes correspond to the degrees from greatest to lowest. Two nodes with the same degree, namely, node 3 and node 9, were chosen to demonstrate the influence of their local clustering coefficients of neighbors. As expected, nodes with a high degree also have a high total C. However, it does not occur on all nodes. For instance, node 136 with degrees 17 and node 17 with degrees 30 have the same total C. Nodes 95 and 48 have a lower total clustering coefficient than node 136 due to their degrees 23 and 20. This measure incorporates both degrees and the local clustering coefficient. Thus, nodes with the same degree do not always have the same premium as those in Table 2 or prior studies by Xu and Hua (2019) [19] and Antonio and Indratno (2021) [26].

**Table 4:** Ten nodes were chosen from a total of 143 nodes. They were selected based on their degree and uniqueness of behavior.

Nodes	105	17	95	48	136	3	9	24	86	42
Degree	42	30	23	20	17	12	12	8	6	1
C	0.1452	0.1793	0.1779	0.2316	0.625	0.8182	0.4545	0.3929	0.6000	0.0000
Total	16.3043	10.7722	9.5123	8.6777	10.7032	7.4761	4.9562	3.2678	3.3778	0.0000
Quadratic	37.0036	26.7478	19.6042	17.0074	12.1189	8.8299	10.4092	6.9031	4.4710	1.0000
Linear	25.6957	19.2278	13.4877	11.3223	6.2968	4.5239	7.0438	4.7322	2.6222	1.0000
Exponential	16.6983	13.0673	8.7503	7.2212	3.7442	2.5877	4.4903	3.0576	1.6296	1.0000

At various rates, the inhibitory action reduces the effectiveness of infections. Quadratic functions have the highest overall value, followed by linear and exponential functions. Policy underwriters can choose these functions based on indications of cybersecurity or network requirements. For instance, the speed of data transmission is decreasing if they have a long route. To obtain a more accommodating premium for network features, we use the function resulting in a more realistic premium change than would be obtained without the clustering structure component.

The premium simulation results and 95% confidence intervals for each of the ten selected nodes are shown in Table 5. Additionally, high-degree nodes pose a high threat. The most expensive premium is provided by node 105, which has the highest degree 42. The premium associated with the clustering coefficient demonstrates a shift by offering a lower price. Three functions quadratic, linear, or exponential are all adaptations of the function, with the faster-shrinking function resulting in reduced premiums. At nodes 3 and 9, the importance of the results is immediately apparent. Both nodes have a degree of 12. Without regard for the clustering arrangement, these two nodes offer the identical premium of 2.9. (currency unit). However, after adapting to the clustering structure of its neighbors, node 3 provides a lower price. These findings are consistent with the fact that node 3 has a lower total clustering inhibition function than node 4. This approach is successful because it takes the metric under consideration so that the premium is dependent on both the degree and the clustering structure.

**Table 5:** The premium of the ten selected nodes. Premiums and confidence intervals (CI 95%) for selected nodes without clustering functions, and with clustering functions (quadratic, linear, exponential).

Node	Without Clustering Coefficient	With Clustering Coefficient		
		Quadratic	Linear	Exponential
105 CI 95%	4.4023 (4.4665,4.338)	4.3534 (4.4175,4.2894)	4.0036 (4.0644,3.9428)	3.4862 (3.5393,3.4331)
17 CI 95%	3.8224 (3.8836,3.7612)	3.6982 (3.7557,3.6406)	3.4808 (3.5363,3.4253)	3.0197 (3.0728,2.9666)
95 CI 95%	3.4922 (3.5495,3.4348)	3.3992 (3.4538,3.3446)	3.1268 (3.1788,3.0748)	2.8463 (2.8963,2.7963)
48 CI 95%	3.3690 (3.4255,3.3124)	3.2858 (3.3399,3.2317)	3.0008 (3.0519,2.9496)	2.7055 (2.7554,2.6556)
136 CI 95%	3.1914 (3.2447,3.1381)	2.8747 (2.924,2.8254)	2.5193 (2.5662,2.4724)	2.3981 (2.4437,2.3526)
3 CI 95%	2.9031 (2.9554,2.8507)	2.6198 (2.6674,2.5723)	2.2919 (2.3371,2.2466)	2.2573 (2.3033,2.2113)
9 CI 95%	2.8968 (2.8684,2.7654)	2.8169 (2.8684,2.7654)	2.5770 (2.6265,2.5275)	2.3756 (2.4206,2.3306)
24 CI 95%	2.6989 (2.7491,2.6486)	2.6404 (2.6884,2.5925)	2.4842 (2.5311,2.4372)	2.4076 (2.455,2.3601)
86 CI 95%	2.4968 (2.5442,2.4494)	2.4179 (2.4642,2.3717)	2.3690 (2.4161,2.3219)	2.2534 (2.2982,2.2085)
42 CI 95%	2.3162 (2.3619,2.2704)	2.2901 (2.3361,2.244)	2.2856 (2.331,2.2402)	2.2602 (2.3043,2.2161)

The premium with the quadratic function produces a minor change, whereas the exponential function produces the most difference. Additionally, the resultant premium supports the overall result of the neighbor clustering function (TN), which lowers as the function becomes quicker. The total clustering inhibition function of neighbors, which combines the degree and local clustering coefficient, is the crucial metric of a network for calculating the premium with this approach.

**Fig 12:**
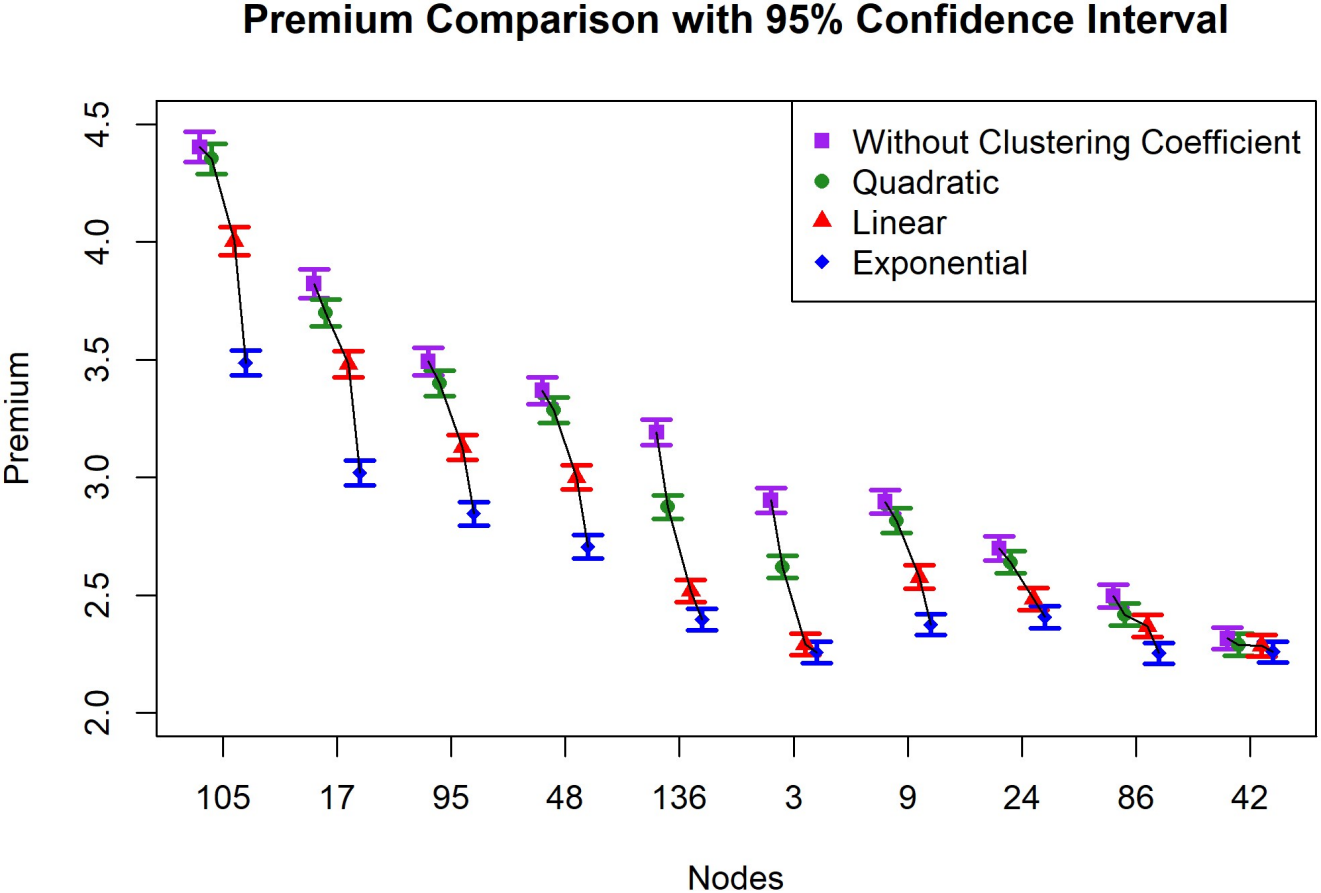
Premium comparison for ten selected nodes. Confidence interval plot with 95% CI without and with clustering coefficients.

Fig 12 illustrates the premium findings in the confidence interval plot. The top position of each node is always determined by the premium without the clustering coefficient, followed by the quadratic inhibition function. The exponential gives the most change of premiums. The nodes have been arranged according to their degree. In general, there are still impacts of degree, although this is not the only impact. At nodes 9 and 3, which have the same degree, both premiums and improvements using the clustering effect are different.

Additionally, the figure depicts how premium fluctuations become more significant as risk grows. The disparity between premiums with and without clustering coefficients is more critical at node 105 with the highest premium than at other nodes with lower premiums. When applied to very high-risk instances, this condition requires an adjustment factor to guarantee that the premium remains enough to cover future risks.

**Fig 13:**
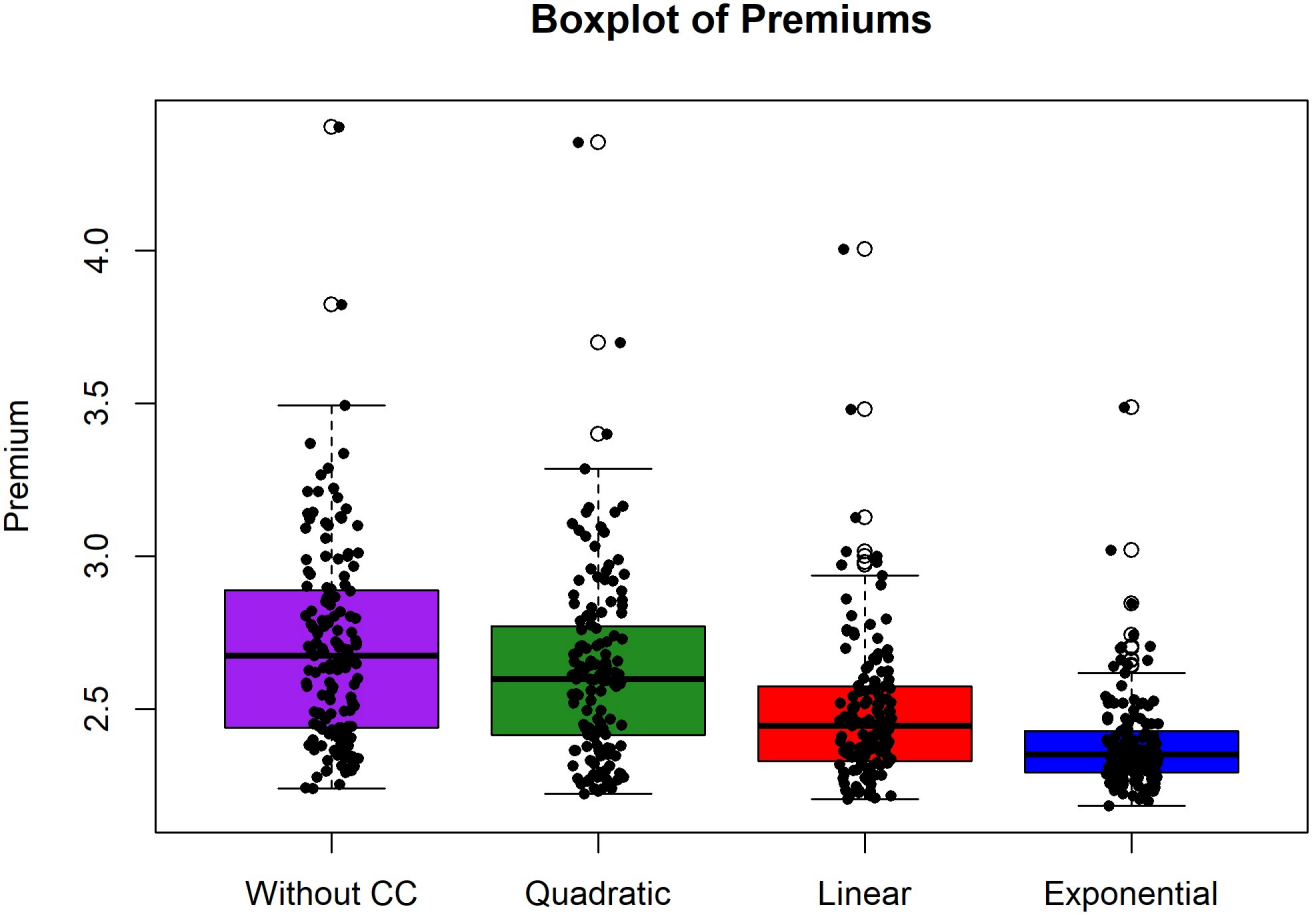
Boxplot of premium comparison of control variables (without CC). Boxplot considers without CC and with CC (quadratic, linear, exponential).

We provide premiums for 143 nodes to confirm the overall results. Premium boxplots of 143 nodes without and with clustering coefficients are shown in Fig 13. The boxplot findings corroborate the evidence of an improvement in the premium price model with the clustering structure. Each range of boxplots decreases when the model without CC is replaced with the model with CC using quadratic, linear, and exponential functions. Similarly, an outlier in each boxplot, namely, the best premium, shows that each function decreases.

**Fig 14:**
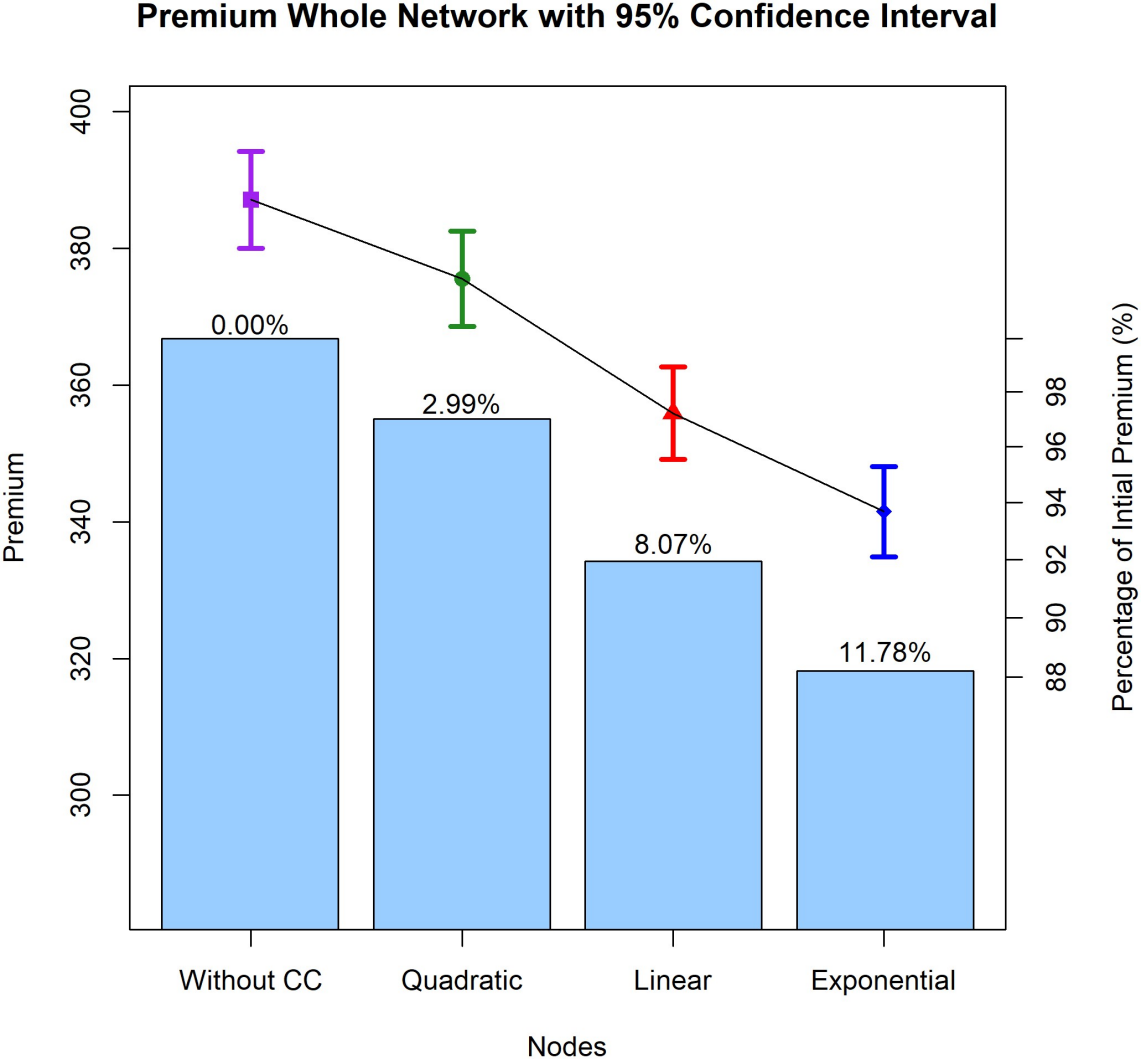
Bar plot and confidence interval plot of premiums for the whole network. Comparison of the total premium (one network premium) in the absence and presence of CC. The text in the bar plot represents the percentage of premium modifications made to the premium without clustering coefficients for each quadratic, linear, and exponential inhibition function.

In aggregate, Fig 14 is a combination of a confidence interval plot and a bar plot depicting a network’s premiums (a total of 143 nodes). These findings also corroborate earlier findings that the presence of a clustering structure might lower premiums. With clustering coefficients, the premiums for quadratic, linear, and exponential functions fell by 2.99%, 8.07%, and 11.78%, respectively. Thus, the overall premium generated by the inhibition function is lower than the premium without the clustering structure.

**Fig 15:**
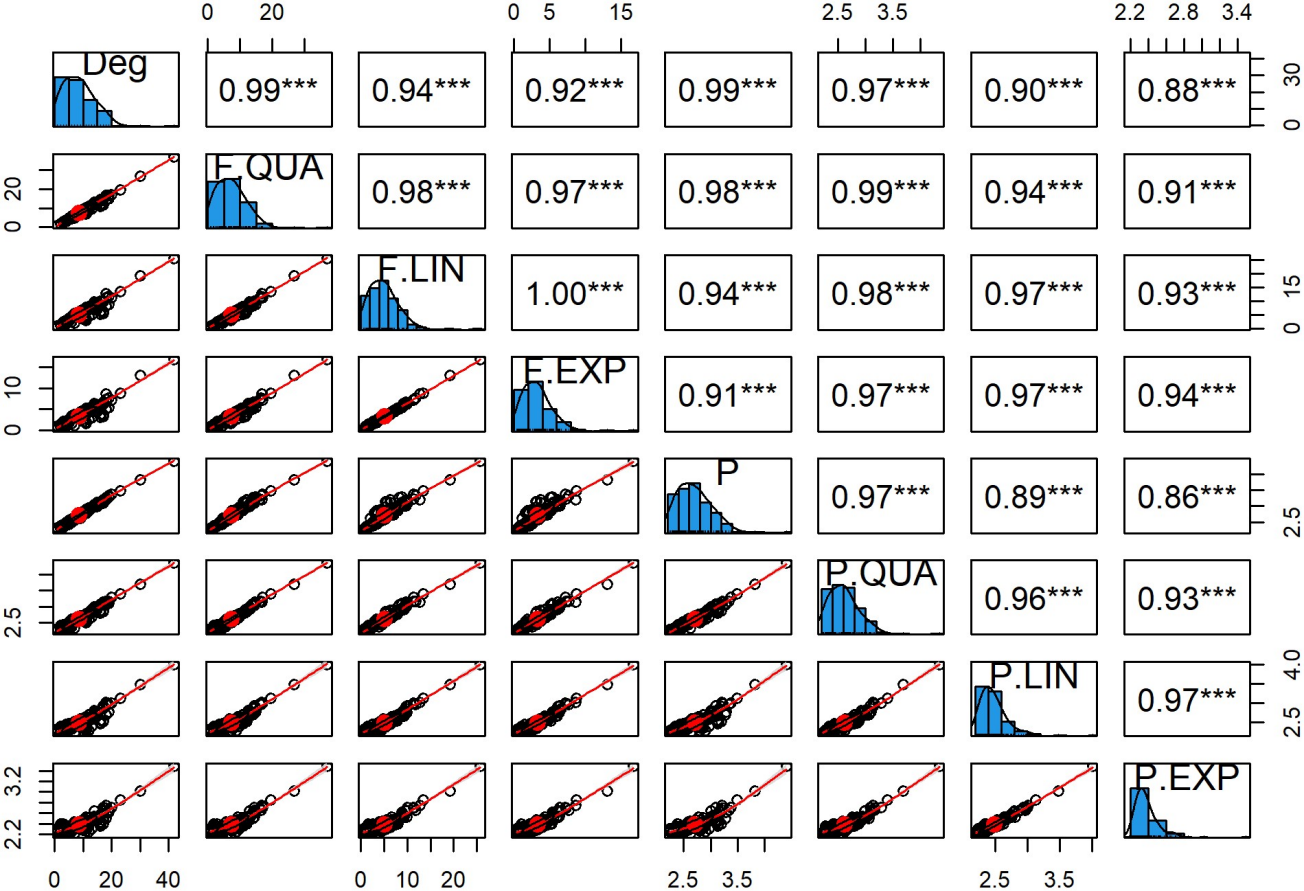
Premium correlation plot. Correlation between degrees (Deg), the total local clustering coefficient (Total C), the premium without CC (P), the premium with a quadratic function (P. QUA), the premium with a linear function (P. LIN), and the premium with an exponential function (P.EXP).

Fig 15 shows the linear correlation between degrees (deg), the total clustering coefficient functions of neighbors (F. QUA, F. LIN, F. EXP), the premium without clustering coefficient (P), the premiums with a linear function (P. LIN), a quadratic function (P. QUA), and an exponential function (P.EXP). Degree, F. QUA, F. LIN, and F. EXP are highly correlated because they are the sum of local clustering coefficients from neighboring nodes. The distinction is in the scale of the adjacency matrix of the model. The value is now between zero and one (in the range of C and *f*(*C*)). The correlation between premiums is extremely strong, with values greater than 0.9. Premiums with local clustering coefficients are used to compensate without clustering coefficients. The correlation between premiums and degree (Deg), F. QUA, F. LIN, and F. EXP decreased from P, P. QUA, P. LIN, and P. EXP sequentially. The more quickly the clustering function decays, the stronger the connection between the premium and the inhibition function. This result means that the inhibitory function chosen affects this relationship.

## Conclusion

We have introduced a modified Markov-based model with a clustering structure factor in the network for premium calculations. To validate the findings, we conducted two types of experiments: regular and real networks. Additionally, theories on regular networks have been established to verify that clustering coefficients influence regular networks. Without the impact of clustering coefficients and a homogeneous rate, each node generates an equal premium. The epidemic inhibition factor was multiplied by the local clustering coefficient to modify the infection rate. As a result, this approach can provide premiums that vary depending on the inhibition function employed, which can be quadratic, linear, or exponential. The results are also significant in large networks (real networks). The correlation between the total inhibitory function and the premiums is stronger than that between the degree and the premiums. Thus, this approach calculates the premium more comprehensively since it considers two network properties, namely, the degree and the local clustering coefficient.

Our novel technique can minimize the premium depending on the features of clustering. These findings corroborate Wu and Liu (2008) [32] and Bo and Song et al. (2017) [33], who found that the clustering coefficient decreases the efficacy of epidemic transmission. This element has been effectively integrated into the premium calculation. By giving a more realistic premium based on the clustering structure, this suggested technique can improve the Markov-based model developed by Xu and Hua (2019) [19] and Antonio and Indratno (2021) [26]. Thus, the flexibility of the proposed approach in application enables it to provide premium improvements that are not homogenous (overestimate) and are more suitable. The limitation is the inclusion of a single element impacting the efficacy of the epidemic. Indeed, the model may incorporate a wide range of other variables. Another limitation is that each node continues to perform the same function. The inhibitory properties of each node may vary.

Future research should explore the usage of diverse functions at each node. The clustering coefficient metric as a function of communication weights may be a critical element to consider in determining how epidemics spread [44] in future studies. Complexity in large-scale simulations encourages the creation of more efficient algorithms, such as a modification of the Gillespie algorithm [35]. From the perspective of mathematical modeling, the theory and application of fractional differential equations [45] to risk modeling [46] or mixed fractional risk processes [47], particularly cyber risk, might be an attractive research area. Epidemic modeling in combination with fractal theory or sets [48] is also required to give a novel viewpoint on understanding viral transmission dynamics [49] for predicting cyber insurance claims.

## Supporting information

S1 Data (XLSX)
